# Unraveling function and diversity of bacterial lectins in the human microbiome

**DOI:** 10.1038/s41467-022-29949-3

**Published:** 2022-06-03

**Authors:** Louis J. Cohen, Sun M. Han, Pearson Lau, Daniela Guisado, Yupu Liang, Toshiki G. Nakashige, Thamina Ali, David Chiang, Adeeb Rahman, Sean F. Brady

**Affiliations:** 1 Department of Medicine, Icahn Genomics Institute, Icahn School of Medicine at Mount Sinai, New York, NY USA; 2 Rockefeller University, New York, NY USA; 3 Laboratory of Genetically Encoded Small Molecules, Rockefeller University, New York, NY USA; 4 Division of Internal Medicine-Pediatrics, University of Massachusetts Medical School, Worcester, MA USA; 5 Human Immune Monitoring Core, Icahn School of Medicine at Mount Sinai, New York, NY USA

**Keywords:** Microbiology, Computational biology and bioinformatics

## Abstract

The mechanisms by which commensal organisms affect human physiology remain poorly understood. Lectins are non-enzymatic carbohydrate binding proteins that all organisms employ as part of establishing a niche, evading host-defenses and protecting against pathogens. Although lectins have been extensively studied in plants, bacterial pathogens and human immune cells for their role in disease pathophysiology and as therapeutics, the role of bacterial lectins in the human microbiome is largely unexplored. Here we report on the characterization of a lectin produced by a common human associated bacterium that interacts with myeloid cells in the blood and intestine. In mouse and cell-based models, we demonstrate that this lectin induces distinct immunologic responses in peripheral and intestinal leukocytes and that these responses are specific to monocytes, macrophages and dendritic cells. Our analysis of human microbiota sequencing data reveal thousands of unique sequences that are predicted to encode lectins, many of which are highly prevalent in the human microbiome yet completely uncharacterized. Based on the varied domain architectures of these lectins we predict they will have diverse effects on the human host. The systematic investigation of lectins in the human microbiome should improve our understanding of human health and provide new therapeutic opportunities.

## Abstract (web summary)

Lectins are non-enzymatic carbohydrate binding proteins important to human cellular functions. Here, the authors characterize a lectin produced by a human associated bacterium, and show that interacts with myeloid cells in the blood and intestine, suggesting commensal microbiota lectins as a diverse and widespread mechanism to interact with host physiology.

## Introduction

The human microbiome is believed to be important to human health and if perturbed can perpetuate disease^1–3^. There are many mechanisms through which the microbiota affects its human host although only a few have been explored in detail. In a shift from traditional DNA sequencing studies of the human microbiome, we utilize functional metagenomic screening methods and human stool samples to identify microbiota genes that are predicted to encode for diverse mechanisms by which microbes could perturb human physiology^4,5^. One commensal bacterial effector gene (Cbeg) family that we found in multiple patient stool samples is composed of uncharacterized genes predicted to encode for lectins (e.g., *Cbeg4* and *Cbeg5*). Lectins are produced by almost all forms of life as a means of mediating cell interactions using proteins and carbohydrate ligands^6–9^. Glycans are common components of extracellular matrices and as such lectin mediated recognition of cell surface glycans is frequently part of how an organism finds a niche, evades host defenses or protects against pathogens^7,8^. Human lectins have a broad range of functions across diverse cell types including the regulation of the microbiome^10–15^. Lectins have also been developed therapeutically as antibiotics, vaccine adjuvants and for the treatment of cancer among other indications^15–17^. Despite the importance of human lectins to cell signaling and microbiome interactions, studies of microbial lectins have largely overlooked human commensal organisms and focused almost exclusively on pathogens^18^.

Here, we report on the bioactivity of a prevalent commensal organism encoded lectin through the study of the metagenome derived Cbeg5 lectin. Using in vitro and in vivo systems, we demonstrate that Cbeg5 selectively activates human myeloid cell populations including monocytes, macrophages and dendritic cells. Interestingly, Cbeg5 conveys distinct patterns of host interaction based on cell type, body site location and cell differentiation. Furthermore, our systematic analysis of human commensal bacteria genomes and metagenomes revealed thousands of uncharacterized lectin genes that are highly prevalent in individuals, distributed throughout the human microbiome, and found in almost all genomes from human-associated bacteria. The large number of unknown and diverse lectin genes encoded by the human microbiota suggest that the Cbeg5 lectin discussed here may be part of a much larger network of host-microbial interactions mediated by lectins. We believe that lectins encoded by human microbiota represent a biologically relevant, functionally diverse and yet largely unstudied effector family. The systematic investigation of these lectins will improve our understanding of how the human microbiome contributes to health and disease.

## Results

### Cbeg4 and Cbeg5 are lectins

**Fig. 1:**
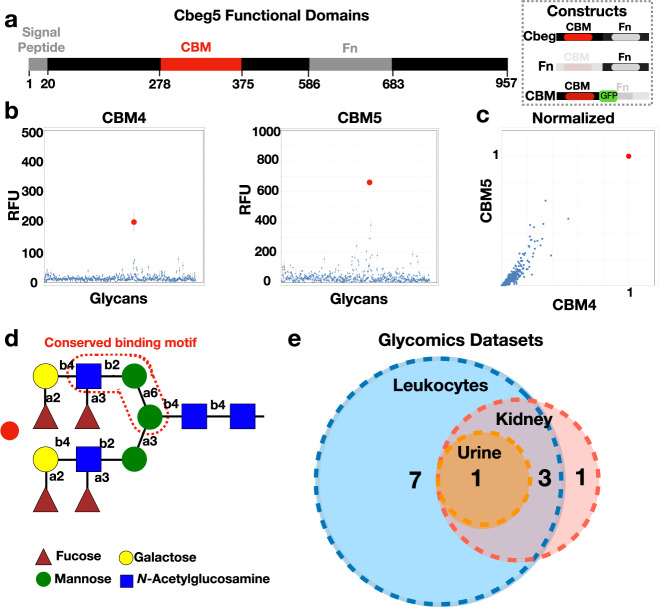
Cbeg4 and Cbeg5 glycomics. **a** Cbeg5 functional domains (InterPro) and protein constructs created for bioactivity assays (box). **b** Cbeg4 and Cbeg5 protein constructs were generated containing only the CBM domain and a GFP tag. The Cbeg5 CBM protein (CBM5) and the Cbeg4 CBM protein (CBM4) were assessed in a binding assay against a panel of 609 N-linked and O-linked glycans (Functional Glycomics Consortium). CBM5 and CBM4 were assayed at 5 μg ml^−1^ and 50 μg ml^−1^ in 6 replicates and glycan binding quantified as relative fluorescent units (RFU). Dot plots of CBM4 and CBM5 glycan binding screens at a concentration of 5 μg ml^−1^ (mean ± s.e.m). **c** Plot of CBM4 vs CBM5 glycan binding normalized to the top glycan binder (set at RFU = 1). Protein concentration is 50 μg ml^−1^. **d** The structure of the top bound glycan (red dot in **b**, **c**) is pictured. Analysis of CBM4/CBM5 bound and unbound glycans identifies a conserved binding motif outlined in red dotted line (glycopattern). **e** A Glyconnect search of glycomics datasets for samples containing the conserved N-linked glycan motif was performed. Glycan structures containing the conserved binding motif were most commonly identified in human leukocyte datasets (11/12 glycan structures). Four glycan structures were shared by human leukocyte, urine and kidney datasets. One glycan structure was exclusive to a kidney dataset.

A predicted domain analysis of *Cbeg4* and *Cbeg5* revealed both genes encode proteins containing a secretion signal peptide, a fibronectin type 3 domain (Fn; IPR003961) and a carbohydrate-binding module domain (CBM, CBM6-CBM35-CBM36_like_2, IPR033803) (Fig. 1a)^4^. Fn domains are common to eukaryotic and bacterial proteins reflecting a shared evolutionary history; however, the function of the Fn domain remains unclear^19^. In eukaryotes, Fn domains are non-specifically distributed in 2% of all proteins whereas, in bacteria, Fn domains are commonly seen in extracellular glycohydrolases. Carbohydrate-binding domains facilitate interactions between proteins and carbohydrates as part of carbohydrate-active enzymes or non-enzymatic carbohydrate-binding proteins (e.g., lectins, sugar transporters)^20,21^. The presence of a secretion signal and a carbohydrate-binding domain together in the absence of an enzymatic domain suggests the proteins encoded by *Cbeg4* and *Cbeg5* may directly impart their biological activity by binding to an extracellular carbohydrate (i.e., a secreted lectin). Among 865 proteins found in the Uniprot database that are predicted to contain a CBM6-CBM35-CBM36_like_2 carbohydrate-binding domain we identified 108 different domain architectures and only 2 functionally characterized proteins (Uniprot, Interpro). The two functionally characterized proteins with a CBM6-CBM35-CBM36_like_2 domain are enzymes and not lectins; one is a xanthan lyase isolated from *Bacillus sp*. *GL1* and the other is a golgi trafficking enzyme (golvesin) isolated from *Dyctostelium discoideum*^22,23^. Analysis of the Cbeg4 and Cbeg5 protein sequence by SWISS-MODEL suggests Cbeg4 and Cbeg5 exist as monomeric proteins. The domain architecture of Cbeg4 and Cbeg5 is specific to commensal *Bacteroides* species and is distinct from any functionally characterized lectins (Supplementary Data 1).

To determine the carbohydrate-binding specificity of Cbeg4 and Cbeg5 we screened each carbohydrate-binding domain protein for binding against an array of diverse carbohydrate structures including N and O-linked glycans (Consortium for Functional Glycomics). Full-length Cbeg4 and Cbeg5 GFP fusion proteins were insoluble when expressed in *E. coli*. Soluble material for use in the glycan screen was obtained by subcloning each carbohydrate-binding domain as a GFP fusion protein (Fig. 1 box)^21^. Cbeg4 and Cbeg5 carbohydrate-binding domains (CBM4, CBM5) showed similar binding patterns against a panel of 609 naturally occurring and synthetic glycans (Fig. 1b–d). In fact, for both CBMs the same glycan produced the highest signal in the carbohydrate-binding assay (Fig. 1d). Glycan structures that bound to either CBM4 or CBM5 share a common Galβ1–3GlcNacβ1–2Manα1–3Man motif (Glycopattern, Fig. 1d). In human glycomics data from the Glyconnect database, this N-linked glycan sub-structure is most frequently seen in datasets generated from peripheral blood mononuclear cells (PBMC) (Fig. 1e)^24–27^. Based on their domain content and glycan-binding properties Cbeg4 and Cbeg5 appear to be lectins that bind leukocyte-associated N-linked glycan motifs.

### Cbeg5 activates myeloid immune cells

**Fig. 2:**
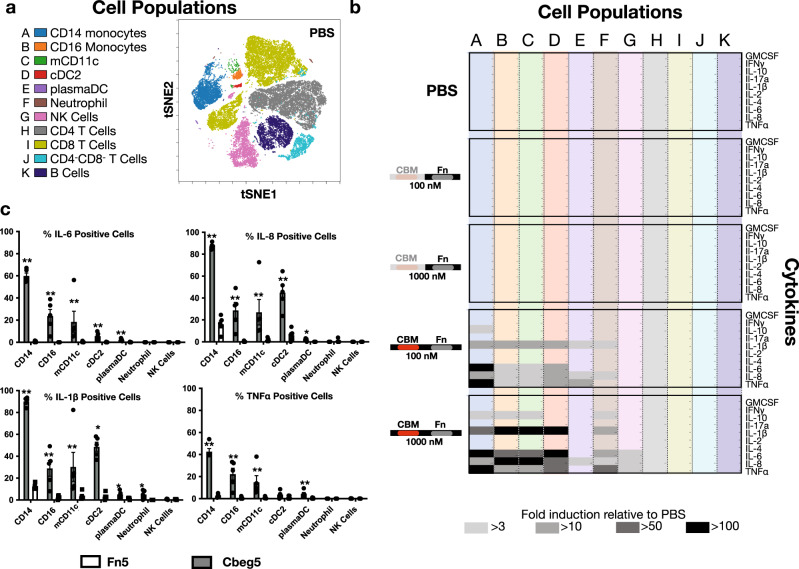
PBMC response to Cbeg5. Fresh PBMC were isolated from healthy patients and exposed to Cbeg5, Fn5 and PBS for 6 h and analyzed by CyTOF with a panel of 16 cell surface and 10 cytokine markers. **a** Cell surface markers distinguish major immune cell populations (A–K) that correlate to groups identified by tSNE analysis (Supplementary Fig. 2). **b** Fold induction of 10 cytokines across cell populations A–K from (**a**). Fold induction is calculated relative to PBS control. Cytokine responses > 3-fold to > 100-fold are marked with gray-shaded boxes. Proteins assayed at 100 nM or 1000 nM as indicated. (Data is a single experiment. PBS data is normalized to a replicate experiment with PBS). **c** Bar graph (mean ± s.e.m) of percent positive cells for each cytokine in specific cell populations in response to Cbeg5 or FN3 control at 500 nM concentration (*N* = 5 from three independent experiments, unpaired, two-sided Mann–Whitney test comparing Cbeg5 to Fn3 control in each cell population) ***p < 0.001 **p < 0.01 *p < 0.05.

Leukocyte responses to these lectins were explored using PBMCs collected from healthy patients and two distinct Cbeg5 protein constructs. These included full-length Cbeg5 and the Cbeg5 Fn domain alone (Fn5) (Fig. 1 box). PBMCs were co-cultured for 6 h with each recombinant protein and analyzed by mass cytometry (CyTOF) using antibodies to 16 cell surface markers and 10 cytokines (Supplementary Table 1). Supervised clustering of the 16 cell surface markers resolve leukocytes into known populations that correlate well with clustering by unsupervised t-distributed stochastic neighbor embedding (viSNE) (Fig. 2a, Supplementary Fig. 1).

For CD14^+^ monocytes, CD16^+^ monocytes, and cDC2 dendritic cells Cbeg5 increased IL-1β, IL-6, IL-8, IL-10 and TNFα in a dose-dependent manner (Fig. 2b, c)^28,29^. Cbeg5 also affected cytokine production in CD1c^−^CD14^−^CD16^−^CD11c^+^ myeloid cells (CD3^−^CD19^−^CD56^−^CD66^−^HLADR^+^), which we will refer to as myeloid CD11c+ (mCD11c). These cells may represent cDC1 dendritic cells though additional markers would be required for a definitive categorization. The induction of cytokines in these four cell populations was striking with a >100-fold increase relative to the PBS control. There was a smaller cytokine response in plasmacytoid dendritic cells, neutrophils and NK cells, and there was no detectable response to Cbeg5 in T cells or B cells. Among all cell populations the induction of cytokines was the most potent and widespread in CD14^+^ monocytes affecting >90% of the cells even when exposed to as little as 100 nM of purified protein (Fig. 2b, c). Fn5 alone did not elicit a cytokine response above that observed for the vehicle control (PBS) in any cell population suggesting that the cytokine induction activity of Cbeg5 is conferred by its CBM domain. The strong bioactivity of Cbeg5 against CD14^+^ monocytes at 100 nM is consistent with the bioactivity of Cbeg5 in the glycan-binding screen which performed at 46 and 466 nM concentrations.

To assess early PBMC responses we exposed PBMCs to Cbeg5 for 20 min and repeated the CyTOF experiment using the original panel of 16 cell surface markers and an additional panel of 8 antibodies targeting phosphorylated proteins (phospho-proteins) in diverse signaling pathways^30^. When Cbeg5 exposed PBMCs were clustered using either supervised or unsupervised methods they organized into the same cell populations as they did in the cytokine assay (Supplementary Fig. 2). Relative to the PBS control, Cbeg5 induced at least a twofold increase in phospho-proteins in CD14^+^ monocytes, CD16^+^ monocytes, mCD11c and cDC2 populations (Supplementary Fig. 2). In contrast to the increase in cytokines we observed for plasmacytoid DCs, neutrophils or NK cells, in the 20 min co-incubation experiment Cbeg5 did not affect signaling pathway markers in these cell populations. This suggests that early cell signaling events may proceed through pathways not measured by this panel or that the observed cytokine responses are indirectly mediated. Among all cell populations Cbeg5 had its greatest effect on signaling pathways in CD14^+^ monocytes, which parallels our observations in the cytokine assay (Supplementary Fig. 2). Fn5 failed to elicit a significant response beyond what was seen for the PBS control in any cell population. Both CyTOF studies indicate that Cbeg5 specifically affects myeloid immune cells and that this effect is the greatest for CD14^+^ cell populations.

### Tissue-specific Cbeg5 activity

**Fig. 3:**
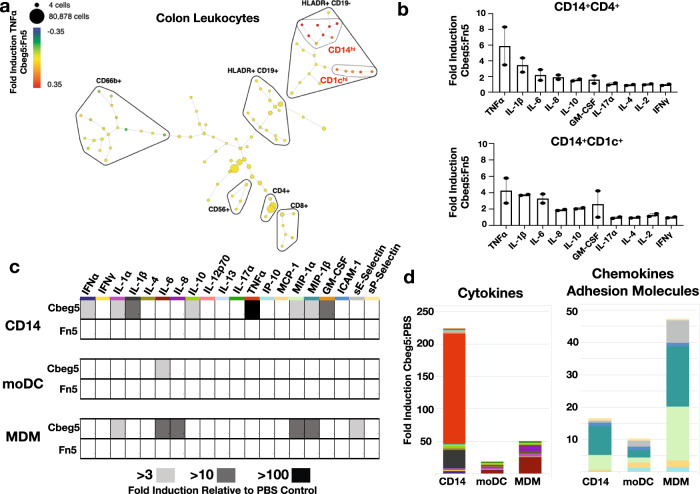
Intestinal leukocyte response to Cbeg5. Fresh colon tissue was obtained from patients at the time of surgery for isolation of lamina propria leukocytes (LPL). **a** LPL cells were exposed to Cbeg5 or Fn5, analyzed by CyTOF and the data evaluated by SPADE. A SPADE plot for Cbeg5 induced TNF-α production from intestinal leukocytes. Fold induction calculated relative to Fn5. Cbeg5 induces cytokine production in two myeloid cell clusters (CD19^−^CD56^−^CD66b^−^HLADR^+^) that are CD14^hi^ or CD1c^hi^. Clusters are annotated by manual gating of indicated cell surface markers. **b** Cells from the CD14 cluster and CD1c cluster were analyzed to identify CD14^+^CD4^+^ cells (monocyte derived macrophage markers) and CD14^+^CD1c^+^ cells (monocyte derived dendritic cell markers). Bar graphs depict Cbeg5 induced cytokine production in CD14^+^CD4^+^ and CD14^+^CD1c^+^ cells (mean ± s.e.m, *N* = 2, two independent experiments). **c** Isolated blood CD14^+^ monocytes were differentiated in vitro into macrophages (MDM) and dendritic cells (moDC) and exposed to 500 nM concentrations of Cbeg5, Fn5 and PBS. Cytokines, chemokines, growth factors and adhesion molecules were analyzed by Luminex (Procarta Plex^TM^). Heatmap of cytokine production with > 3-fold to > 100-fold induction shown with gray shaded boxes (induction relative to PBS control, data is mean of triplicate experiments for CD14^+^ monocytes and duplicate experiments for mMAC, mDC). **d** Bar graph of cytokine or chemokine and adhesion molecule production for cell populations in response to Cbeg5. Bar graph colors are derived from labels used in the heatmap (**c**).

As *Cbeg5* was isolated from a stool metagenomic library it is expected to have arisen from a bacterium associated with the gastrointestinal tract^4^. Cbeg5 produced by intestinal bacteria could contact blood leukocytes either by transport across the epithelial barrier or by disruption of the epithelial barrier during inflammation^31^. It could also directly affect immune cells at the mucosa, especially macrophages and dendritic cells that sample luminal antigens^32–34^. Intestinal leukocytes are distinct from peripheral leukocytes in particular in their responses to known bacterial effectors^35–37^. We therefore used immune cells isolated from colonic tissue to examine Cbeg5’s effect on intestinal leukocytes. Lamina propria leukocytes (LPL) isolated from fresh colon samples were exposed to either full-length Cbeg5 or Fn5 alone for 6 h. We then carried out a CyTOF study using the same cell surface and cytokine markers as we used to analyze PBMCs. A SPADE (Spanning-tree Progression Analysis of Density normalized Events) analysis of treated LPLs identified a number of cell populations that showed increased cytokine production in response to Cbeg5^38^. Cbeg5 increased TNFα and IL-1β levels at least threefold relative to Fn5 treated cells in two branches on the SPADE tree (Supplementary Fig. 3, Fig. 3a). Cell surface markers associated with these two branches identify them collectively as myeloid cells (CD19^−^CD56^−^CD66^−^HLADR^+^) with high expression of CD1c (CD1c^hi^ cluster) in one branch and CD14 (CD14^hi^ cluster) in the other branch (Fig. 3a, Supplementary Data 4). The same analysis run on LPLs isolated from a second patient specimen showed Cbeg5 induced cytokine production in cell populations on the same branches of the SPADE tree and in the same CD1c and CD14 clusters (Supplementary Fig. 3). In the second patient, we isolated a sufficient number of leukocytes to add a third treatment condition of PBS alone. In this case, we observed no difference in cytokine induction between Fn5 and the PBS control (Supplementary Fig. 3). Manual gating of intestinal leukocytes confirms Cbeg5 induced cytokine production in CD1c^+^ and CD14^+^ cell populations (Supplementary Figs. 1 and 3.) As observed with Cbeg5 activation of blood leukocytes, Cbeg5 activation of intestinal leukocytes is specific to myeloid cells; however, the magnitude of Cbeg5 induced cytokine production for intestinal myeloid cells is much less than that observed in blood myeloid cells.

Cbeg5 activates CD14^+^ myeloid cell populations found in the blood as well as the intestine. Blood CD14^+^ monocytes traffic to the intestine where they differentiate into dendritic cells (monocyte-derived dendritic cells; moDC) and macrophages (monocyte-derived macrophages; MDM)^32^. Interestingly, CD14^+^CD1c^+^ (moDC markers) and CD14^+^CD4^+^ (MDM markers) cell populations from the intestine were activated to a lesser extent than blood CD14^+^ monocytes (Fig. 3b). To determine whether the difference in Cbeg5 activation of CD14^+^ populations in the blood and intestine was intrinsic to cellular differentiation we isolated blood CD14^+^ monocytes and in vitro differentiated them into MDM and moDC. CD14^+^ monocytes as well as in vitro differentiated moDCs and MDMs were exposed to Cbeg5, Fn5 or PBS for 6 h and culture supernatants were then analyzed by Luminex technology for cytokine production (Procarta Plex^TM^ Human Inflammation Panel). In all three cell types Cbeg5 increased cytokine production relative to PBS, while Fn5 was indistinguishable from the PBS control (Fig. 3c). CBeg5 induced cytokine production was the greatest for CD14^+^ monocytes. The largest observed increases were in TNFα (168-fold) and IL-1β (28-fold). By contrast moDC and MDM had a more limited cytokine response to Cbeg5 with the largest increase being for IL-6 (Fig. 3d). In addition to cytokines, the Luminex assay measures chemokines, adhesion molecules and growth factors. Contrary to its effect on cytokine induction, Cbeg5 had its most significant effect on chemokine production for MDMs (MIP-1α, MIP-1β) (Fig. 3d). Cbeg5 also selectively increased the production of the adhesion molecule sE-selectin and the growth factor GM-CSF for MDMs and for CD14^+^ monocytes, respectively (Fig. 3d). The distinct activation patterns of CD14^+^ cell populations based on cellular differentiation indicate that Cbeg5 may differentially affect immune cell populations in the blood (CD14^+^ monocytes) and in the intestine (moDCs, MDMs).

### Expression of Cbeg5 in mice

**Fig. 4:**
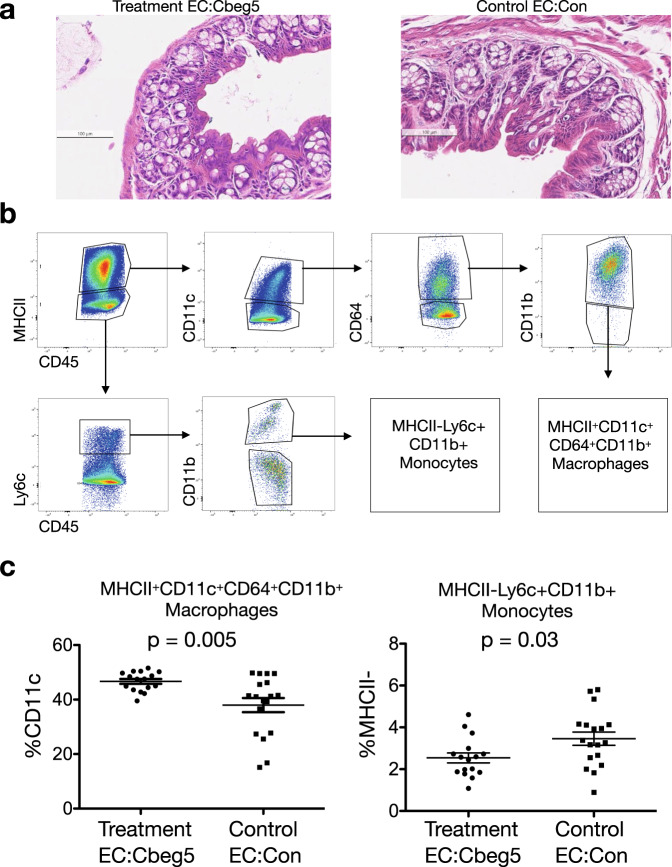
Immune response to Cbeg5 expression in vivo. Wild-type 8-week C57BL/6 mice were colonized with *E. coli* engineered to express *Cbeg5* (Treatment, EC:Cbeg5) or *E. coli* with an empty vector (Control, EC:Con). After 1 week of colonization the colon was analyzed for inflammatory changes by histology (**a**) and for change in immune cell populations by flow cytometry (**b**, **c**). **a** No difference was observed in colon histologic inflammatory indices (one representative image, Supplementary Fig. 5). **b** After gating for singlets and live cells (Aqua LIVE/DEAD) the gating schema is shown for identification of macrophages MHCII^+^CD11c^+^CD64^+^CD11b^+^ and monocytes MHCII^-^Lyc^+^CD11b^+^. **c** There was a significant increase in lamina propria MHCII^+^CD11c^+^CD64^+^CD11b^+^ macrophages and a decrease in MHCII^−^Lyc^+^CD11b^+^ monocytes in the treatment group (*N* = 16 (8 M/8 F) Treatment, *N* = 18 (9 M/9 F) Control. Data represents 4 independent experiments. Error bars are mean ± s.e.m. Control and treatment populations compared using an unpaired, two-sided Mann–Whitney test).

The activation of intestinal and blood myeloid cell populations by Cbeg5 suggested that in gastrointestinal tract bacterial expression of this human-microbial-lectin could affect the host immune system. To explore this possibility, 8-week old C57/BL6 mice were colonized with *Escherichia coli* engineered to express *Cbeg5* under the control of its native promoter (EC:Cbeg5, treatment group) or *E. coli* transformed with an empty vector (EC:Con, control group). In the previous PBMC and intestinal leukocyte experiments, Cbeg5 induced distinct responses in peripheral and intestinal myeloid cell populations suggesting either the potential for causing inflammation or for regulating immune cell differentiation and trafficking. As the peripheral immune response was profoundly inflammatory we first aimed to assess whether colonization with bacteria expressing *Cbeg5* would cause colitis. After 1 week of colonization, mice were examined for evidence of colon inflammation by clinical and histologic endpoints. There was no significant difference between treatment groups in weight gain, food or water intake, and neither group developed diarrhea by examination of fecal pellets (Supplementary Fig. 4). When sections of paraffin embedded colon tissue stained with hematoxylin and eosin were scored for inflammation no differences were observed between treatment groups (Fig. 4a, Supplementary Fig. 4).

Without clinical or histologic evidence of inflammation we examined whether expression of *Cbeg5* might lead to changes in mucosal immune cell populations. One week post colonization LPLs were isolated from the colons of colonized mice and analyzed by flow cytometry using antibody panels that distinguish myeloid cell populations (MHCII, CD64, Ly6C, CD11c, CD11b, CD45) from T cell populations (CD45, CD4, RORγt, FOXP3, GATA3) (Supplementary Table 1). There were no significant changes between groups in examined CD4^+^ T cell populations (Supplementary Fig 5), whereas among myeloid cell populations there was a significant increase in MHCII^+^CD64^+^CD11b^+^CD11c^+^ cells and a significant decrease in MHCII^-^Ly6c^+^CD11b^+^ cells in the mice colonized with bacteria that express *Cbeg5* (Fig. 4b, c). MHCII^+^CD64^+^CD11b^+^CD11c^+^ are macrophage markers and MHCII^-^Ly6c^+^CD11b^+^ are monocyte markers^32,33,39–41^. The observed increase in macrophage populations is consistent with our previous in vitro experiments that showed an increase in macrophage cell trafficking chemokines, growth factors and adhesion molecules in response to Cbeg5.

### Human-microbial-lectins are diverse and unknown

Using publicly available human microbiome and lectin datasets (Human Microbiome Project, Unilectin) we sought to understand the prevalence of genes that encode lectins across individuals and body sites^3,42^. Our analysis of human microbiome sequencing data identified genes with significant sequence similarity (BLASTP, >90% sequence similarity) to 20 well-characterized lectins (Supplementary Table 2). Thirteen of these are adhesins from *E. coli* and the remaining are toxins, nutrient transporters or adhesins from *Streptococcus sanguinis, Streptococcus mitis, Mycobacterium bovis, Helicobacter pylori* and a bacteriophage. These well-characterized lectins are encoded by a small subset of human microbiome samples and are largely associated with pathobionts or pathogens (Supplementary Fig 6). In contrast, *Cbeg4* and *Cbeg5* have no significant sequence similarity to any previously characterized lectin genes in Unilectin, are highly prevalent across human microbiome samples and are associated with common commensal species (Supplementary Fig 6). As the study of bacterial lectins has traditionally focused on pathogens, it is not surprising that a search of human microbiome samples using functionally characterized lectins identified genes that largely encode for toxic effector functions in low prevalence pathobionts. Our functional characterization of Cbeg4 and 5 as lectins from common human-associated bacteria and the lack of sequence similarity to previously characterized lectins led us to explore whether the human microbiome encodes additional common uncharacterized lectins that may be unique to this environment and plays additional uncharacterized roles in microbiota host signaling (i.e., human-microbial-lectins).

**Fig. 5:**
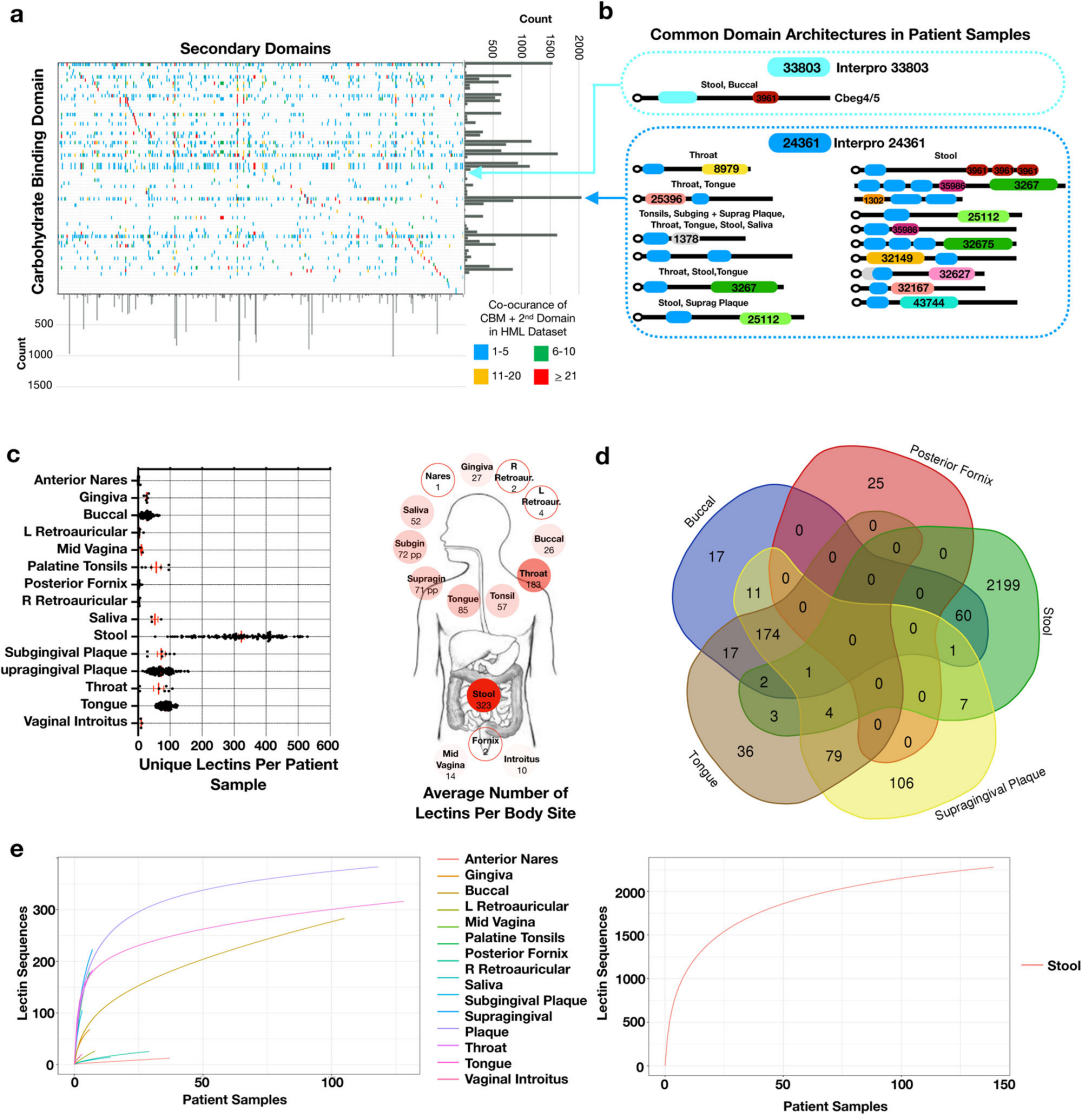
Human-microbial-lectin prevalence and diversity. **a** All domains present in the human-microbial lectin dataset are organized based on co-occurrence between a carbohydrate-binding domain and secondary domains in the same lectin. Bar graphs summarize the count of each domain in the dataset and box color reflects the frequency of domain co-occurrence. **b** Lectin domain architectures containing the Cbeg4/5 carbohydrate-binding domain (IPR33803) and the Bacteroidetes-Associated Carbohydrate-binding Often N-terminal domain (IPR024361) are pictured. IPR0244361 is the most common domain in the human-microbial-lectin dataset and present in 891 lectins. The most prevalent lectins (top 10% for each body site) containing these domains are pictured. For IPR33803 only the Cbeg4/5 domain architecture is identified in abundance in patient samples. **c** prevalence of human-microbial-lectins in patient samples from the Human Microbiome Project. Red bar is mean ± SEM. Summary of the average number of human-microbial-lectins per person for each body site is pictured. Color of circle is proportional to the mean lectin count with stool set as 100%. **d** Overlap of lectin genes between five body sites. **e** Rarefaction analysis of human-microbial-lectin genes for each body site based on number of samples from the Human Microbiome Project. Stool is pictured on a separate plot due to the large number of lectin sequences. Human digestive tract image in **c** is from the National Institute of Diabetes and Digestive and Kidney Diseases media library.

To identify additional uncharacterized lectins encoded by the human microbiota, we aligned predicted proteins from the Human Microbiome Project (HMP) to proteins in the Uniprot database with unknown functions and at least one predicted carbohydrate-binding domain but no catalytic domain (Supplementary Table 3)^43,44^. Microbiome sequences that aligned at ≥90% sequence identity to a Uniprot entry were considered potential human-microbial-lectins. To focus our analysis on uncharacterized lectin sequences we removed protein sequences predicted to have functions related to carbohydrate transport, cell structure or proteins with known functions such as flagella, fimbriae, or adhesins. The resulting collection of predicted and uncharacterized human-microbial-lectins contains 5517 unique protein sequences with 1149 bioinformatically predicted domain architectures composed of 74 distinct carbohydrate-binding domains and 460 secondary domains (Fig. 5a, Supplementary Data 2).

The most common carbohydrate-binding domain found in this dataset of uncharacterized proteins is the Bacteroidetes-Associated Carbohydrate-binding Often N-terminal domain (IPR024361) (Fig. 5a, b). This domain is present in 891 human-microbial-lectin sequences that are associated with 124 distinct domain architectures. IPR024361 has not yet been assayed broadly for glycan-binding though it is believed to bind a mucin glycan^45–47^. As mucins are a structurally diverse family of glycoproteins that are commonly produced by the intestine it is not unexpected that a carbohydrate-binding domain which targets mucins is the most common domain present among human-microbial-lectins and is associated with a large diversity of domain architechtures^48,49^. In contrast, the carbohydrate-binding domain from Cbeg4 and 5 (IPR033803) is present in a much smaller number of predicted lectins (16) and is associated with many fewer domain architectures (6) (Fig. 5a, b). Among proteins found in abundance in human microbiome sequencing datasets, the IPR033803 domain is only associated with those that contain Cbeg4 and 5-like domain architectures. In addition, in an analysis of all IPR033803 containing proteins deposited in NCBI, Cbeg4 and 5-like domain architectures were only encoded by common commensal *Bacteroides* sp. (Supplementary Data 1). These observations suggest that Cbeg4 and 5 and more generally *Bacteroides* sp. with lectins that have the same domain architecture may have evolved specific and conserved roles in host microbiota signaling.

We examined the prevalence and distribution of uncharacterized human-microbial-lectins across individuals, body sites and human-associated bacteria reference genomes using HMP datasets^43,44^. Uncharacterized human-microbial-lectins are highly prevalent among patients, found in every sampled body site and present in 99% of reference genomes (Fig. 5c, Supplementary Fig 7, Supplementary Data 3). Within the human microbiome, the stool metagenome has the largest number of uncharacterized lectin sequences (2278) with each patient’s stool containing on average 323 unique lectin genes. In general, body sites from the gastrointestinal tract have more unique lectin sequences then body sites from the urogenital tract or skin. Only 18% of predicted lectin sequences are shared between two or more microbiome sites (Fig. 5d, Supplementary Tables 4 and 5). Oral microbiome sites share a large percentage of lectin sequences whereas the stool and the posterior fornix are composed almost entirely of lectins specific to those sites (95% stool, 88% posterior fornix). Among the large number of predicted lectin sequences and domain architectures we identified in stool samples they range across individuals from being highly prevalent like Cbeg4 and 5 (>90% of samples) to being quite rare (<10% of samples) (Supplementary Fig 8).

When taken together our analyses of predicted lectin genes in the HMP data, suggests that compared to well-characterized lectins from bacterial pathogens, uncharacterized lectins encoded by the human microbiota like Cbeg4 and Cbeg5 are highly prevalent in the human microbiome and are likely to encode a diversity of functions. The identification of lectin genes that are specific to body sites and individuals as well as genes that are shared across individuals may indicate lectins facilitate both highly conversed as well as more variable microbiome associated functions. A rarefaction analysis of lectin gene sequences from each body site suggests that while additional sequencing of some sites, especially the urogenital tract and skin, is unlikely to uncover a significant number of new uncharacterized lectins, other sites, like most sites in the oral microbiome are predicted to contain significant additional diversity outside of that already captured in existing HMP datasets (Fig. 5e).

## Discussion

Cbeg5 is part of a large collection of predicted human-microbial-lectins that are distinct from lectins isolated from bacterial pathogens, almost entirely uncharacterized and highly prevalent across individuals. The characterization of Cbeg5’s activity against myeloid cells in the blood and gastrointestinal tract suggests a potential role for human-microbial-lectins in governing commensal interactions and not simply in driving local inflammatory responses. The exact mechanisms through which Cbeg5 elicits a response in myeloid cells will require further investigation including determining the nature of the target antigen(s) and how expression of *Cbeg5* in vivo affects the host over longer colonization times in disease models. The widespread prevalence of *Cbeg5* and related sequences in human microbiome samples as well as the observed effects of Cbeg5 on myeloid cells from different body sites suggest Cbeg5-like lectins may be key regulators of immune homeostasis.

The identification of a commensal bacterium encoded lectin that interacts with human myeloid cells is reminiscent of observations made in other organisms where lectins have been found to facilitate mutualistic host-microbe interactions^6–9^. Interestingly, in a parallel fashion human myeloid cells use lectins to mediate interactions with commensal organisms^7,8,12,50^. For example, myeloid cells use C-type lectins such as the macrophage mannose receptor, dendritic cell receptors DC-SIGN or CLEC9A and Dectin-1 on monocytes to maintain microbiome homeostasis^51^. Dysregulation of human lectin-mediated host-microbial interactions have been linked to the development of diseases associated with the microbiome including cancer, inflammatory bowel disease and diabetes among others^8,51–54^. The fact that both the human microbiota and the human immune system produce lectins that are capable of interspecies interactions adds to the growing examples where the host and the microbiota appear to use related signaling systems and metabolites for communication^55–57^.

Based on the numerous domain architectures we identified in predicted lectins from the human microbiome, human-microbial-lectins are likely to be more functionally diverse than their well characterized counterparts described from bacterial pathogens. To date the role of lectins in the human microbiome has focused on their function as adhesins or in the binding and transport of glycans for bacterial metabolism. In our analysis we excluded lectins that are clearly associated with these well-characterized functions. Our functional analysis of Cbeg5 suggests the diversity of lectin functions in the human microbiome may extend to regulation of the mucosal immune system and that the systematic exploration of human-microbial-lectin functions in the human microbiome could provide another axis for elucidating the mechanistic details of how the microbiota contributes to human health and disease. The functional characterization of lectins from the human microbiota and the engineering of microbes to produce these lectins may prove to be a productive strategy for modulating human health. Lectins derived from other organisms are already being developed to treat and diagnose human autoimmune, infectious and malignant diseases^15^. Whether human-microbial-lectins can be harnessed as therapies will be interesting to investigate going forward.

## Methods

### Subcloning pET28c-His_6_-Cbeg5^21–957^ (Cbeg5, Fn5)

Subcloning primers with overhanging NdeI and XhoI sequences (Supplementary Table 6) were used to amplify the nucleotide sequence for each construct from the pJWC1-Cbeg5 (4L05) template vector following a standard PCR protocol for Q5 High-Fidelity DNA Polymerase (New England Biolabs). The annealing temperature was set to 67 °C, and the extension time was set to 90 s for Cbeg5. The annealing temperature was set to 68 °C, and the extension time was set to 30 s for Fn5. The amplified gene sequences were digested with NdeI and XhoI (New England Biolabs), ligated into NdeI- and XhoI-digested pET28c using T4 DNA ligase (New England Biolabs), and transformed into electrocompetent *E. coli* EC100 cells. Single colonies were inoculated into 50 ml LB medium with 50 µg ml^−1^ kanamycin. The cultures were miniprepped, and the identity of the plasmid confirmed by Sanger sequencing.

### Subcloning pET28c-His_6_-GFP Cbeg4-CBM and Cbeg5-CBM (CBM4, CBM5)

Subcloning primers with overhanging NotI and XhoI sequences (Supplementary Table 6) were used to amplify the nucleotide sequence of the CBM domain of *cbeg5* (*cbeg5-CBM*) from the pJWC1-cbeg5 (4L05) template and the CBM domain of *cbeg4* from the pJWC1-cbeg4 (33g04) template. All templates were amplified by following a standard PCR protocol for Q5 High-Fidelity DNA Polymerase (New England Biolabs). The amplified gene sequences were digested with NotI and XhoI (New England Biolabs), ligated into the NdeI- and Xho1-digested vector pET His6 GFP TEV LIC cloning vector (Addgene https://www.addgene.org/browse/sequence/13778/) and transformed into electrocompetent *E. coli* EC100 cells. Single colonies were inoculated into 50 ml LB medium with 50 µg ml^−1^ kanamycin. The cultures were miniprepped, and the identity of the plasmid was confirmed by Sanger sequencing.

### Overexpression and purification of protein variants

Plasmids were transformed into *E. coli* BL21(DE3) T7 Express cells (New England Biolabs), and glycerol freezer stocks were prepared from single colonies. Freezer stocks were inoculated into 50 ml LB with 50 µg ml^−1^ kanamycin, and cultures were grown overnight at 37 °C, 200 rpm (New Brunswick Innova 44). Overnight cultures were subinoculated (1:100 dilution, 10 ml) into 1 L lysogeny broth (LB) with 50 µg ml^−1^ kanamycin. The resulting cultures were incubated at 37 °C, 200 rpm until OD_600_ ≈ 0.6, induced with 0.5 mM IPTG, and incubated at 18 °C, 200 rpm for 20 h (New Brunswick Innova 44). The cells were pelleted at 4200 × g for 20 min at 4 °C, resuspended with 50 ml LB, and transferred to 50 ml Falcon tubes. The cells were pelleted again, and after the supernatant was discarded, they were stored at –80 °C. The weight of each wet cell pellet was approximately 2 g.

Protein purifications were typically conducted at 2 L scales, i.e., combining two 1 L cell pellet per prep. All steps were conducted in aplementary cold room or on ice. Cells were thawed on ice and resuspended with 100 ml lysis buffer. The lysis buffer contained 50 mM KH_2_PO_4_ pH 7.2, 300 mM NaCl, 1 mM PMSF, 0.5% Triton-X, and 25 U Benzonase Nuclease (Millipore Sigma). The cells were lysed on ice by probe sonication using a Misonix Sonicator 3000 or by French press using an Avestin Emulsiflex C5. For sonication, a program following a sequence of 10 s on and 20 s off over 3 min was used. For French press, the culture was passed through the instrument five times. The crude lysate was clarified by ultracentrifugation (80,000 rcf, 15 min, 4 °C) using a Optima L-100 XP Ultracentrifuge (Beckman Coulter). The soluble lysate was transferred to 50 ml Falcon tubes.

Ni-NTA resin (5 ml; Thermo Fisher Scientific) was equilibrated with 5 CV of lysis buffer. Soluble lysate was applied to the column. The column was washed with 20 CV of wash buffer (50 mM KH_2_PO_4_ pH 7.2, 300 mM NaCl, and 10 mM imidazole), and the bound protein was eluted with 5 CV of elute buffer (50 mM KH_2_PO_4_ pH 7.2, 300 mM NaCl, and 250 mM imidazole) and collected. The protein (25 ml) was dialyzed using Spectra/Por 12–14 kDa dialysis tubing (Spectrum Labs) against 500 ml PBS pH 7.2, where the buffer was replaced 4 times over 72 h.

The dialysate was transferred to a 50 ml Falcon tube and centrifuged (4200 × *g*, 30 min, 4 °C) to pellet insoluble protein precipitate that formed during dialysis, and the soluble fraction was filtered through a 0.22-µm syringe filter. The protein (25 ml) was concentrated using an Amicon 3 K or 30 K MWCO spin concentrator (Millipore Sigma), depending on the molecular weight of the protein (Sup Table S1), to 3 ml. The protein was further purified on an ÄKTA Purifier FPLC system (GE Healthcare Life Sciences) using an ENrich SEC 650 10 ×300 mm column (Bio-Rad Laboratories) housed at 4 °C. A 0.5 ml volume of protein was injected onto the column. The elution buffer was PBS, and the flow through was 1.0 ml min^−1^. Eluent fractions (0.5 ml) were collected, and those containing purified protein were identified by SDS-PAGE and pooled for final confirmation by LCMS.

To remove possible contaminating endotoxin, the purified protein was treated with Pierce High Capacity Endotoxin Removal Resin (Thermo Scientific) following the manufacturer’s protocol. The protein solution was filtered through a 0.22-µm syringe filter and concentrated. The final concentration of protein typically ranged from 10 µM to 100 µM as determined by the absorbance at 280 nm using a NanoDrop instrument and the extinction coefficient (Supplementary Table S1). Aliquots (25–500 µl) were partitioned into microcentrifuge tubes, flash frozen in liquid nitrogen, and stored at –80 °C. The purity of the protein samples was evaluated by SDS-PAGE and LCMS. All samples were analyzed again for presence of endotoxin by the Pierce high sensitivity chromogenic endotoxin quantification kit and confirmed to be below the limit of detection (0.1 EU/ml). All samples were freeze-thaw cycled once and thawed immediately prior to use.

### Screening of carbohydrate-binding proteins against glycan microarray and bioinformatics analyses

CBM4 and CBM5 were sent to the Consortium for Functional Glycomics for screening against a panel of 609 target glycans including both N-linked and O-linked glycans. (http://www.functionalglycomics.org). Proteins were assayed at 50 μg ml^−1^ and 5 μg ml^−1^ concentrations in 6 replicates. Streptavidin was used as a positive control. Data are presented as relative fluorescent units which are set relative to the background fluorescence. The highest and lowest values are removed and data presented as a mean of the remaining 4 replicates. No pre-screening oligomerization step is performed. The output from the screen was then entered into the Glycopattern program for identification of conserved binding motifs^58^. The top binding motif common to both CBM4 and CBM5 glycan screening was then used to query diverse glycomics datasets using Glyconnect to identify structures from sample tissues, cells or organisms that contain this key motifs (https://glyconnect.expasy.org/about). The Glyconnect database was queried to identify structures that are N glycans with the conserved Galβ1–3GlcNacβ1 determinant.

### Isolation of human PBMC

Fresh PBMC from deidentified healthy patient donors were obtained from the Icahn School of Medicine Human Immunology Monitoring Core or from StemExpress (https://icahn.mssm.edu/research/human immune-monitoring-center, https://www.stemexpress.com). PBMC at the Icahn School of Medicine were isolated from patients under approval of the Mount Sinai Institutional Review Board (IRB) approved protocol #11-00866. All patients provided written informed consent and the authors were not involved in the sampling or consent process. Sex/age of the participants are unknown. Whole blood was drawn into ACD Vacutainer tubes with EDTA then processed within 3 h. Fifteen millilitres of blood is transferred to a 50 ml conical tube then 15 ml PBS is added. 15 ml Ficoll (Sigma) is added separately to a 50 ml SepMate tube to which 30 ml of diluted blood sample is added. The tube is centrifuged for 10 min at 1200 RCF at room temperature. The top plasma and PBMC layer are separated and transferred to a new 50 ml conical then centrifuged at 515 rcf for 10 min at 4 °C. The supernatant is then decanted and the PBMC pellet is resuspended in 5 ml PBS. Cells are then counted by adding 20 μl of AOPI to 20 μl of PBMC solution and counted on a CelloMeter. Cells are centrifuged a second time at 515 rcf for 10 min at 4 °C then resuspended in cold culture media (RPMI 1650 with 10% Fetal Bovine Serum, 1% penicillin–streptomycin, 10 mM l-glutamine and 20 mM HEPES—ThermoFisher) at 1 million cells per 180 μl of media for assay.

### Isolation of human Colon LPL

Fresh colon tissue was obtained at the time of resection under IRB-approved protocol #14-00174. All patients provided written informed consent and the authors were not involved in the sampling or consent process. Sex/age of the participants are unknown. Colon tissue was obtained from patients undergoing a surgical resection for malignancy or diverticular disease. Patients took no immune-suppressing medication, had no known immune disease of the bowel (e.g., inflammatory bowel disease, microscopic colitis) and samples were taken from a healthy area of the resected colon as determined by a pathologist. Using biopsy forceps and dissection scissors the mucosa and submucosa were removed and placed into a 15 ml conical tube filled with 10 ml of dissociation media to remove the epithelial cells (HBSS w/o Ca^++^Mg^++^, 1 M HEPES, 0.5 M EDTA). The tube was incubated at 37 °C with shaking at 100 RPM for 15 min (New Brunswick Innova 44). The solution was then vortexed manually for 30 s and passed through a 70 μM cell strainer. After removal of epithelial cells the solution was transferred to a 15 ml conical tube filled with 10 ml of digestion media (HBSS w/o Ca^++^Mg^++^, 2% FCS, DNaseI 0.5 mg ml^−1^, collagenase IV 1 mg ml^−1^). The solution was then incubated at 37 °C with shaking at 100 RPM for 40 min (VWR Incubating Orbital Shaker). The solution was filtered through a 70 μM cell strainer and the filtrate transferred to a 15 ml conical tube with FACS buffer (DPBS w 3% FBS and 1 mM EDTA). The cells were then centrifuged at 652 RCF at 4 °C for 8 min. The supernatant was decanted and cells resuspended in 1 ml of RBC lysis solution for 1 min. 11 ml of FACS buffer was added and cells again centrifuged at 652 RCF at 4 °C for 8 min. The supernatant was decanted and the cells processed using the Dead Cell Removal Kit per protocol (Miltenyi Biotec). The final cell pellet was resuspended in 500 μl of DPBS and cells counted by Tryptan blue exclusion.

### CyTOF

All PBMC or colon LPL cells were plated 1 million cells per well in a 96 well U bottom plate in 180 μl of media (RPMI 1650 with 10% Fetal Bovine Serum, 1% penicillin–streptomycin, 10 mM l-glutamine and 20 mM HEPES—ThermoFisher). All treatment proteins were prepared in 10× solutions then diluted in 20 μl PBS according to desired final assay concentration and transferred to wells.

For the assessment of phosphorylated signaling proteins by CyTOF PBMC were co-incubated with Cbeg5, Fn5, CBM5 and PBS for 20 min at 5% CO_2_ 37 °C along with Rh103 antibody (Fluidigm). Cells were washed twice with FACS buffer (DPBS w 3% FBS and 1 mM EDTA) and fixed in 1.6% paraformaldehyde for 20 min at room temperature. Cells were then washed twice and diluted in FACS buffer and placed at 4 °C until staining and analysis by CyTOF. For assessment of cytokines by CyTOF PBMC or LPL were co-incubated with Cbeg5, Fn5, CBM5, and PBS for 6 h at 5% CO_2_ 37 °C along with 2 μM monensin (Biolegend). After 6 h Rh103 antibody was added to cells and co-incubated for an additional 20 mi. Cells were then washed with FACS buffer and fixed with 1.6% paraformaldehyde at room temperature. Cells were then washed twice in FACS buffer and placed at 4 °C until staining and analysis by CyTOF.

Prior to CyTOF assays previously optimized antibody mixtures were prepared in cell staining media (CSM, Fluidigm). Antibody lists are outlined in Supplementary Table 1. Each sample was washed and resuspended in 800 μl of 1× Barcode Perm Buffer (Fluidigm Inc.). Compatible Pd-barcodes were thawed, resuspended in 100 μL of 1× Barcode Perm Buffer and added to the samples. Samples were incubated on ice for 30 min then washed in CSM and pooled together. Each barcoded set of samples (corresponding to a single treatment condition) was first resuspended in 100 μL of CSM containing 100 U ml^−1^ heparin (Sigma) to block non-specific MaxPar Antibody binding. A titrated surface antibody panel designed to allow identification of all major immune subsets (Supplementary Table 1) was prepared in an additional 100 μl of CSM, filtered through a 0.1 μm spin filter (Amicon) and added directly to the sample. Samples were stained for 30 min on ice, then washed with CSM and fixed with freshly diluted 2% formaldehyde (Electron Microscopy Sciences) in PBS to cross-link and preserve all surface antibodies. The samples were then washed and permeabilized by the addition of 1 ml of ice-cold 100% methanol, added dropwise while vortexing. Samples were incubated on ice for 30 min (or transferred to −80 °C for long-term storage), after which they were washed twice with CSM and again resuspended in 100 ul of CSM containing 100 U ml^−1^ heparin. A titrated panel of validated antibodies against phospho-protein epitopes or cytokine epitopes were prepared in an additional 100 ul of CSM, filtered through a 0.1 μm spin filter (Amicon) and added directly to the sample and incubated for 30 min on ice (Supplementary Table 1). Samples were then washed with CSM and incubated for 30 min in freshly diluted 2% formaldehyde in PBS containing 0.125 nM Ir nucleic acid intercalator (Fluidigm). The samples were washed and stored as pellets in CSM until CyTOF acquisition.

Immediately prior to acquisition, samples were washed once with PBS, once with deionized water and then counted and resuspended at a concentration of 1 million cells ml^−1^ in water containing a 1/20 dilution of EQ. 4 Element Beads (Fluidigm). Following routine instrument tuning, the samples were acquired on a CyTOF2 Mass Cytometer equipped with a SuperSample fluidics system (Victorian Airships) to facilitate bulk sample acquisitions. Samples were acquired at a flow rate of 0.045 ml min^−1^ and an event rate of < 400 events per second. CyTOF FCS files were first concatenated and normalized using the bead-based normalization tool in the Helios software (Fluidigm), the barcoded samples were automatically deconvoluted and cross-sample doublets were filtered using a Matlab-based debarcoding tool^30^ and the resulting files were uploaded to Cytobank for analysis. Cell events were identified as Ir191/193 positive events, and residual Ce140+ normalization beads were excluded. For intestinal leukocytes the live/dead antibody Rh103 was used to assess viability (Supplementary Fig 1). Analysis of all cell events for the colon leukocyte experiment identified immune cell markers in 98% of cells with >85% viability.

### CyTOF analysis

All analyses of CyTOF samples were performed using Cytobank software. High-dimensional analysis used to map the multi-dimensional data into two-dimensional space include SPADE and viSNE. All high-dimensional analysis were performed on singlets and the data files analyzed together to ensure cell clusters are stable across experiments. In all analyses cells were clustered using surface antibody markers. For SPADE visualization of fold changes between populations the Fn5 treatment condition was set as the baseline sample and the analysis targeted 100 nodes. For viSNE analysis, all samples were set to equal sampling at 30000 events per sample and run per default parameters for the software. Major immune populations were identified based on manual inspection of canonical marker expression patterns. For the SPADE analysis manual gating of canonical marker expression patterns are annotated in Fig. 3a and outlined in Supplementary Fig. 1. For the viSNE analysis manual gating of canonical marker expression patterns are annotated in Fig. 2a and outlined in Supplementary Fig. 1.

### Analysis of CD14^+^ derived populations

Primary human CD14^+^ monocytes, MDM and moDC were purchased from Stem Cell Xpress. Fresh cells were aliquoted 2.5 × 10^5^ cells per well in 96-well U bottom plate with 180 μl of media (RPMI 1650 with 10% Fetal Bovine Serum, 1% penicillin–streptomycin, 10 mM l-glutamine and 20 mM HEPES). All treatment proteins were prepared in 10× solutions then diluted in 20 μl PBS according to desired final assay concentration and transferred to wells with primary cells for 6 h and incubated at 5% CO_2_ and 37 °C. After incubation plates were centrifuged at 515 rcf for 10 min and supernatant was collected. Cell supernatant was immediately processed per protocol using the Procartaplex Human Inflammation Panel (Invitrogen) and analyzed on a Luminex 200. Data were analyzed by the ProcartaPlex Analyst 1.0 software.

### Mouse experiments

All animal procedures were approved by the Institutional Animal Care and Use Committee of Mount Sinai School of Medicine (IACUC-2016-0491). Mice were not randomized prior to colonization but cages were selected at random for treatment groups. Equal numbers of 8-week-old C57BL/6 male and female mice were ordered from Jackson Laboratories and housed at the Mount Sinai Animal Facility. Mice were housed at specified pathogen-free health status in individually ventilated cages at 21–22 °C and 39–50% humidity with a XXX light and 12 h light–dark cycle. After acclimatization, mouse drinking water was supplemented with 500 μg/ml of ampicillin 500 μg/ml for 5–7 days to facilitate high levels of colonization. Drinking water was then changed to tetracycline 15 μg ml^−1^ and mice were gavaged with *E. coli* EC100 transformed with either pJWC engineered to express Cbeg5 under regulation of its native promoter (EC:Cbeg5) or an empty pJWC vector (EC:Con)^4,59^. Ten millilitres of overnight cultures of each bacteria were centrifuged and resuspended in 1 ml of PBS. Each mouse was gavaged with 100 μl of this culture and colonization was confirmed at day 4 by recovery of the gavaged bacteria from mouse feces by plating of feces on LB agar with and without tetracyline 15 μg ml^−1^ then isolation of the EC:Con or EC:Cbeg5 plasmids from individual colonies. There was no difference between treatment groups in the number of colony-forming units in mouse feces (Supplementary Fig 4). End-point experiments were performed after 7–10 days of colonization. Fecal pellets were analyzed for consistency (watery or formed) and food/water intake tracked on a per cage basis. All mice were weighed on arrival to the facility, prior to colonization and at the end of the experiment. Drinking water and food intake was measured per cage.

For analysis of colon tissue and leukocytes all mice were euthanized by cervical dislocation. Abdominal cavities were exposed and the colon was identified and removed. 1 cm pieces of colon were then aliquoted into 10% formalin for analysis by histology or HBSS for isolation of intestinal leukocytes. Histologic analysis was performed by Histowiz including interpretation of pathology and scoring of intestinal inflammation. For isolation of LPL the colon tissue was placed into 10 ml of EDTA solution (HBSS with 10% FBS, 5 mM EDTA, 15 mM HEPES) and incubated at 37 °C with shaking at 150 RPM for 20 min (VWR Incubating Orbital Shaker). The colon tissue was then vortexed for 30 s and minced using dissection scissors. The minced tissue was placed into a new conical with 10 ml of digestion solution (RPMI 1640, 2% FBS, 0.5 mg/ML Collagenase D, 0.5 mg/ml DNase) and incubated at 37 °C with shaking at 150 RPM for 30 min (VWR Incubating Orbital Shaker). Contents were homogenized using an 18G needle then filtered through a 100 μM strainer. Cells were then centrifuged at 515 rcf for 20 min. Supernatant was decanted and cells resuspended in 10 ml RPMI 1640. Cells were then strained again through a 40 μM strainer and pelleted again by centrifugation at 515 rcf for 5 min. The cells were then finally resuspended in RPMI 1640. For analysis by flow cytometry 100 μL of 2.4G2 Fc blocker was added to cells and incubated at 4°C for 10 min. Cells were then centrifuged at 805 rcf for 2 min and resuspended in FACS buffer with antibodies (Supplementary Table 1). Antibodies stained for 30 min protected from light at 4 °C. After staining cells were fixed with 2% PFA for 15 min at room temperature. Cells were then washed with FACS buffer three times and analyzed on the LSRII. For intracellular stains cells were first permeabilized and fixed overnight. Cells were then washed and stained with antibodies in permeabilization buffer. Antibodies were co-incubated for 30 min at 4 °C protected from light, then washed twice in permeabilization buffer and reconstituted in FACS then analyzed on the LSRII Using OneComp Beads in parallel. All samples were analyzed in Flowjo v10.8.1. All mouse model outcomes were analyzed by gender group and there was found to be no significant difference. Statistical tests and graphs were prepared with Prism v9.

### Bioinformatics analyses

Cbeg4 and Cbeg5 gene sequences have been previously deposited at Genbank (accession numbers KT336269.1, KT336270.1). The dataset of characterized lectin protein sequences was downloaded from Uniprot (Unilectin). To generate a dataset of uncharacterized (predicted) lectin proteins from human microbiota we first identified carbohydrate-binding domains in Interpro (Supplementary Table 3). All protein sequences from Uniprot with at least one of the identified Interpro carbohydrate-binding domains were downloaded. From this protein sequence dataset, we removed entries with a catalytic domain as annotated by Uniprot or based on a natural-language search of associated Interpro domain descriptions (https://www.ebi.ac.uk/intenz/rules.jsp). To further focus our dataset on uncharacterized lectin proteins from human microbiota we removed protein sequences predicted to have functions related to carbohydrate transport, cell structure or proteins with known functions such as flagella, fimbriae, or adhesins. The keywords used to remove these protein sequences were: TonB, Transport, Channel, Porin, OmpA, Anchor, Receptor, Dockerin, Cohesin, Flagella, Lamin, Lipoprotein, Secretion, RagB, SusD, ScaA, ScaB, Scaffoldin, Septum, Spore, SusE, Wall, Fimbrial, Fim, FimH, Adhesin, PapG, Membrane, Fimbriae, and Adhesion. Finally, to focus on uncharacterized proteins we removed any protein sequences identified by Uniprot as reviewed. The datasets of characterized and uncharacterized lectin protein sequences were used to query reference genomes from the HMP (https://www.hmpdacc.org/hmp/HMRGD/) and the gene index from patient metagenomic samples across different body sites (https://www.hmpdacc.org/hmp/HMGI/). For the reference genome dataset and metagenomic datasets blast databases were prepared for each body site through makeblastdb. Characterized and uncharacterized lectin sequences were then aligned to blast database through BLASTP. Alignments were summarized into a Boolean matrix and a match was determined at > 90% identity. In the uncharacterized lectin database sequences were clustered at 90% identity through USEARCH to generate a final dataset of unique, uncharacterized (predicted), human-microbial-lectins (Supplementary Data 2)^60^. The body site-specific rarefaction analyses of human-microbial-lectins in metagenomic sequencing datasets were performed through the R package vegan with plots prepared through ggplot2. The Venn diagram generated in Fig. 5 was prepared using the program available at http://bioinformatics.psb.ugent.be/cgi-bin/liste/Venn/calculate_venn.htpl.

### Reporting summary

Further information on research design is available in the Nature Research Reporting Summary linked to this article.

## Supplementary information

Supplementary Information

Supplementary Data 1

Supplementary Data 2

Supplementary Data 3

Supplementary Data 4

Reporting Summary

## Data Availability

Gene accession numbers for cloned genes are Genbank KT336269.1, KT336270.1. Publicly available DNA and RNA datasets analyzed in this study are referenced accordingly and references contain links to datasets available for download. Source data are provided with this paper.

## Source data

Source Data
