# Supplementary material for: Unraveling function and diversity of bacterial lectins in the human microbiome
Source: Nat Commun. 2022 Jun 3;13:3101. doi: 10.1038/s41467-022-29949-3 (PMC9166713; doi:10.1038/s41467-022-29949-3)
Supplement: Supplementary file 1 — Supplementary Information [file 41467_2022_29949_MOESM1_ESM.pdf]

Supplementary Information: Unraveling function and diversity of bacterial lectins in the human microbiome

L Cohen et al.

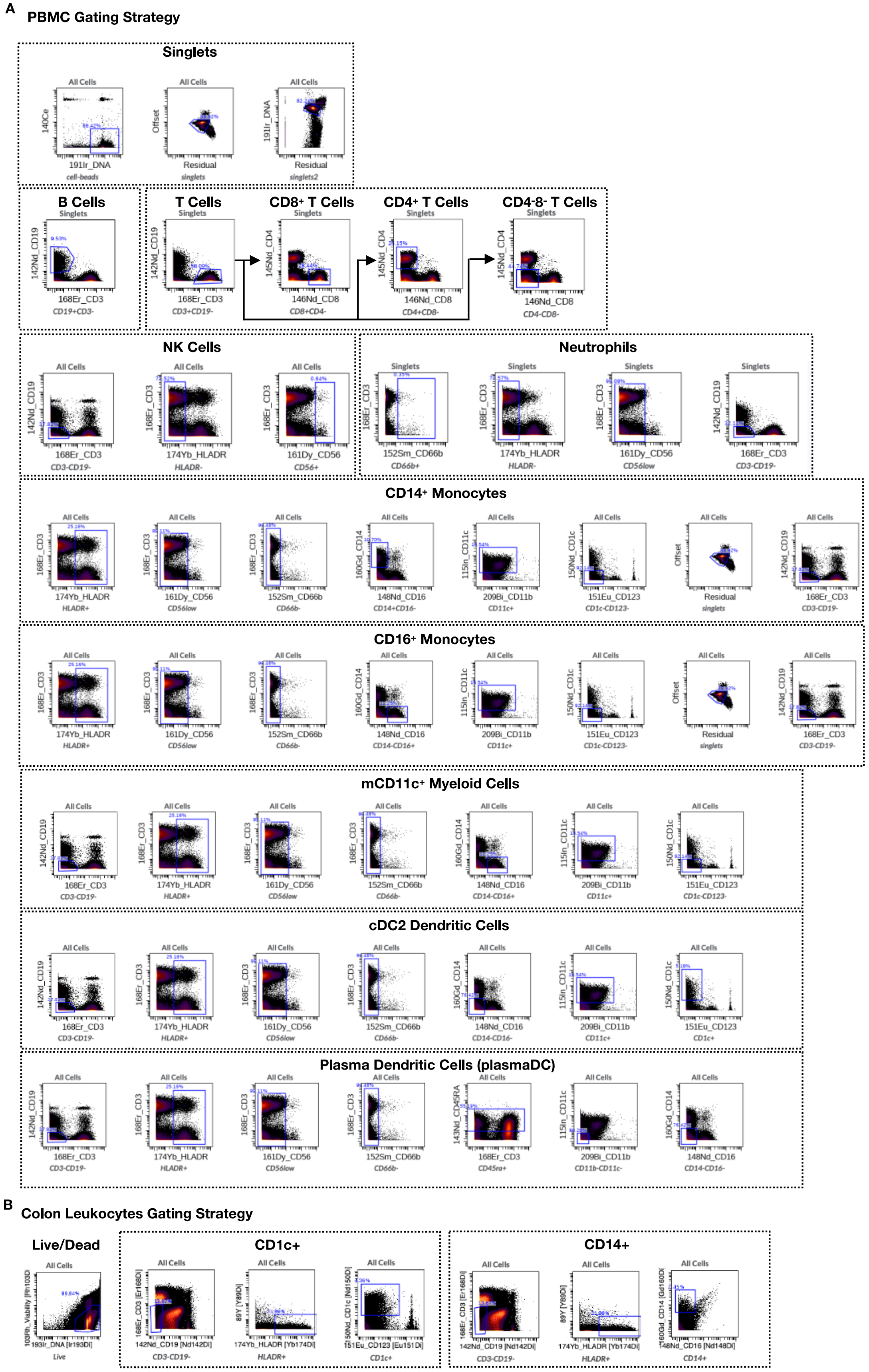

Supplementary Figure 1. Leukocytes analyzed by CyTOF using a panel of 16 cell surface markers. **a,** Gating corresponds to Fig 2 and Extended Data Fig 2. **b,** Gating corresponds to Fig 3 and Extended Data Fig 3

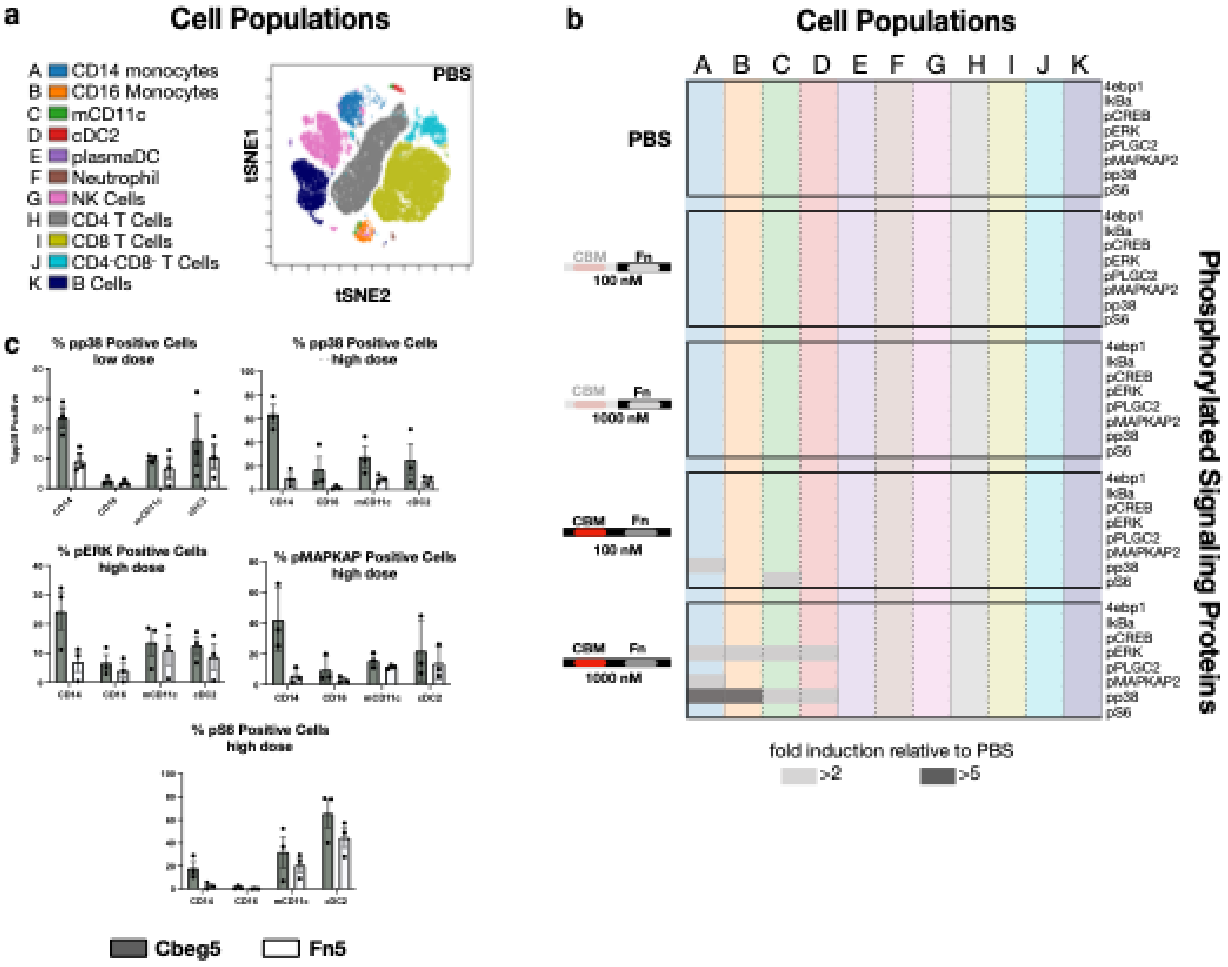

Supplementary Figure 2. Fresh PBMC were isolated from healthy patients and exposed to Cbeg5, Fn5 and PBS for 6h. PBMC were analyzed by CyTOF with a panel of 16 cell surface and 10 cytokine markers. **a,** Cell surface markers distinguish major immune cell populations (A-K) that correlate to groups identified by tSNE analysis (1 representative experiment with PBS). **b,** Fold induction of 8 phosphorylated signaling proteins across cell populations A-K from (**a**). Fold induction is calculated relative to PBS control. Cytokine responses > 2 fold to > 5 fold are marked with grey shaded boxes. Proteins assayed at 100 nM or 1000 nM as indicated. (Data is a single experiment. PBS data is normalized to a replicate experiment with PBS). **c**, Bargraph (mean +/- s.e.m) of percent positive cells for each cytokine in specific cell populations in response to Cbeg5 or Fn5 at low dose (100nM) or high dose (500-1000 nM) concentrations (N = 3 from three independent experiments, unpaired, two sided Mann-Whitney test comparing Cbeg5 to Fn5 for each cell population) p>0.05

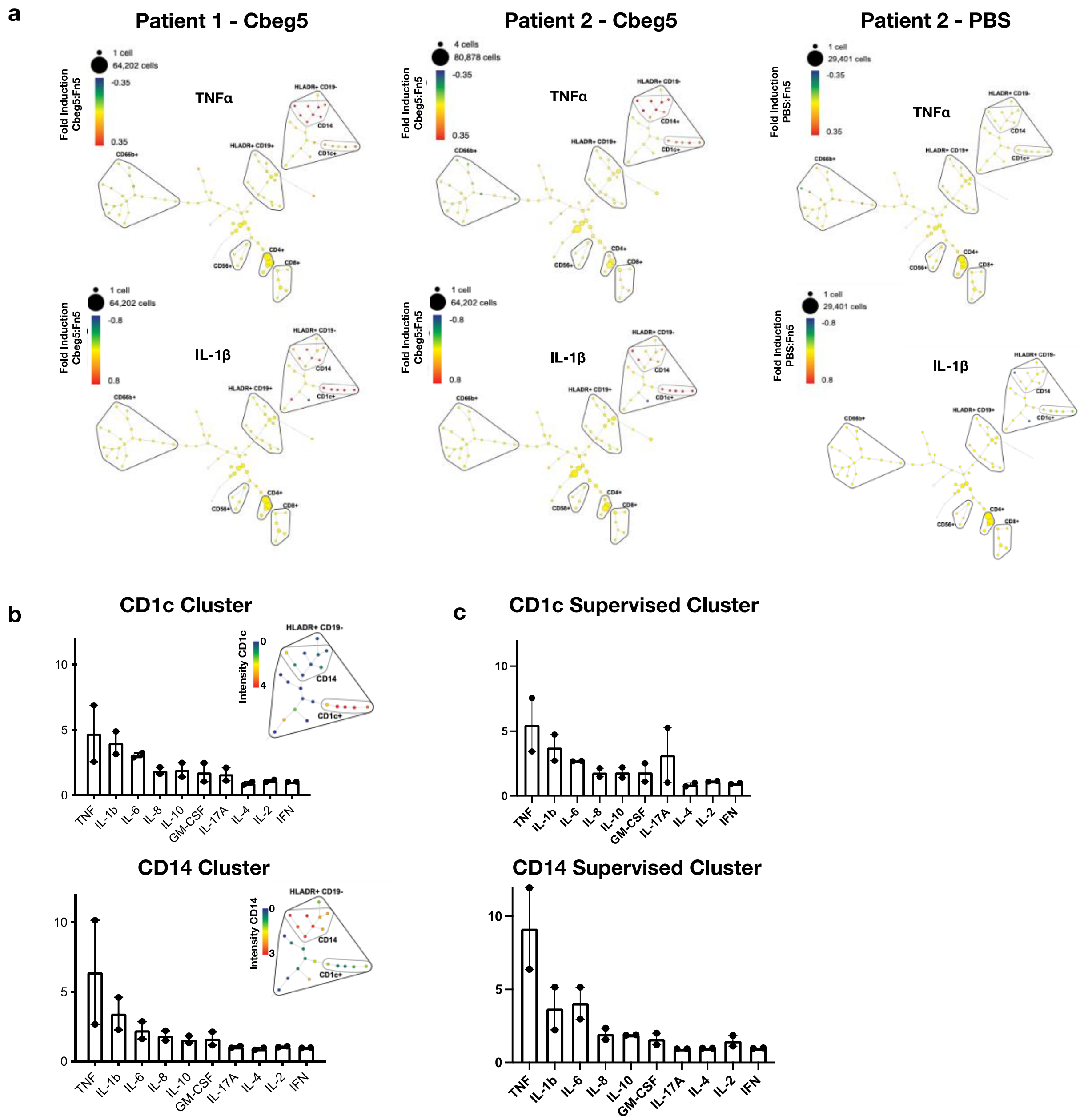

Supplementary Figure 3. Fresh colon tissue was obtained from patients at the time of surgery for isolation of lamina propria leukocytes (LPL). LPL cells were exposed to Cbeg5, Fn5 and PBS for 6 hours then analyzed by CyTOF using the same markers as the PBMC assay **a**, A SPADE plot for Cbeg5 and PBS induced TNF-α and IL-1β from LPL (Cbeg5 - two patient samples, PBS – one patient sample). **b**, Bar graphs of Cbeg5 induced cytokine production from cells in the CD14 cluster (CD14 stain intensity inset) and CD1c cluster (CD1c stain intensity inset) (mean +/- s.e.m, N=2, two independent experiments). **c,** Bar graphs of Cbeg5 incuced cytokine production from CD14+ and CD1c+ cells identified through supervised clustering (Extended Data Figure 1 for gating strategy, mean +/- s.e.m., N=2, two independent experiments).

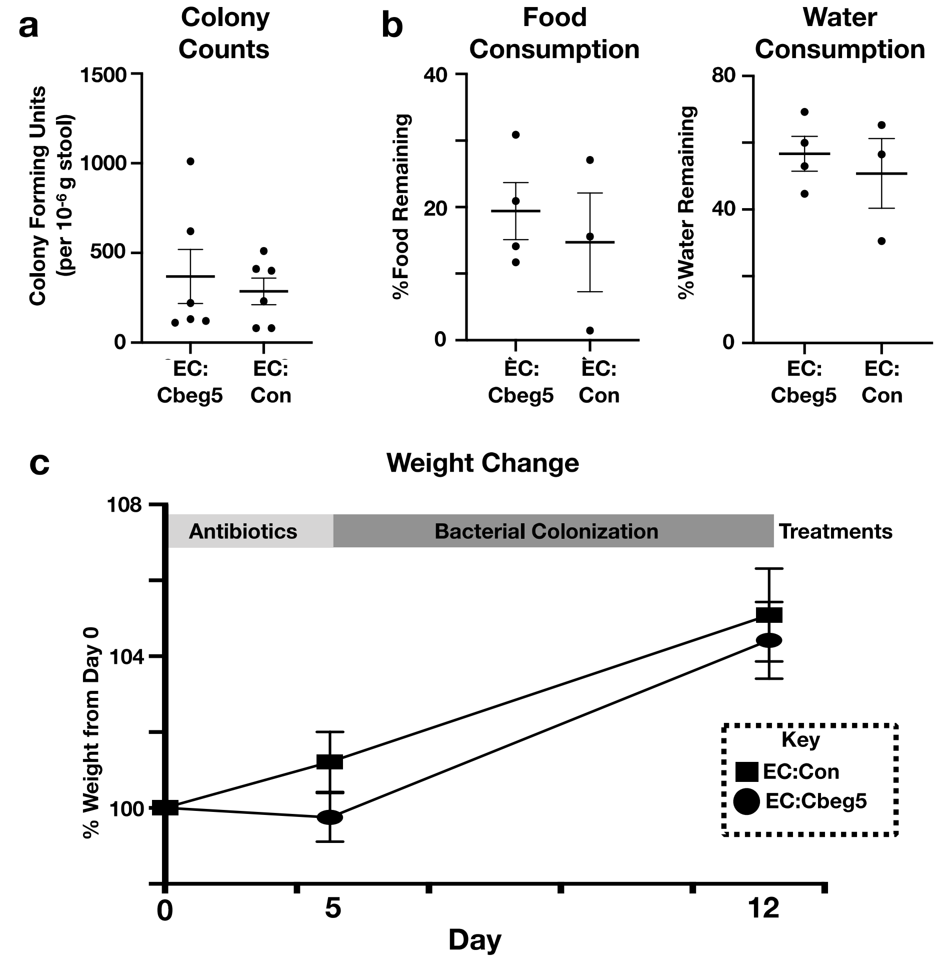

d **Histologic Scoring of Colon Inflammation**

Score 1 Inflammatory Cell Infiltrate				
Severity	Extent	Score	Treatment EC:Cbeg5 (n = 12)	Control EC:Con (n = 9)
None		0		
Mild	Mucosa	1	12	9
Moderate	Mucosa and submucosa	2		
Marked	Transmural	3		

Score 2 Intestinal Architecture				
Epithelial changes	Mucosal architecture	Score	Treatment EC:Cbeg5 (n = 12)	Control EC:Con (n = 9)
Focal erosions		1	12	9
Erosions	± Focal ulcerations	2		
	Extended ulcerations ± granulation tissue ± pseudopolyps	3		

Total Score = Sum of Score 1 + Score 2 (0-6)

Supplementary Figure 4. Wild-type 8 week C57BL/6 mice were colonized with *E. coli* engineered to express *Cbeg5* (Treatment, EC:Cbeg5) or *E. coli* with an empty vector (Control, EC:Con). Mice were treated for 5 days with ampicillin 500 μg ml^{-1} in the drinking water after which mice were gavaged with bacteria and colonized for 1 week. **a,** Analysis of colony forming units for each bacteria (EC:Cbeg5, EC:Con) in stool day 4 after gavage. (N = 6 for each group, unpaired, two-tailed *t*-test p > 0.05) **b,** Food and water consumption was measured from the time of colonization and analyzed on a per cage basis. There was no significant difference in food or water consumption after 1 week between the two groups (N = 4 cages for EC:Cbeg5, N = 3 cages for EC:Con; graphs are mean +/- s.e.m; groups compared by unpaired, two-tailed *t*-test, p > 0.05) **c,** Weight was measured per mouse on arrival to the facility (day 0) after initial antibiotic administration (day 5) and at completion of the study (day 12). There was no significant difference in weight change between the two treatment groups (N = 12 for EC:Cbeg5, N = 9 for EC:Con; graphs are mean +/- s.e.m; groups compared by 2-way ANOVA, p > 0.05 for the trend). **d,** After 1 week of bacterial colonization colon tissue was formalin fixed and sent to Histowiz for analysis by a pathologist. There was no difference between treatment groups for histologic scoring of inflammation (all mice had a total score of 2).

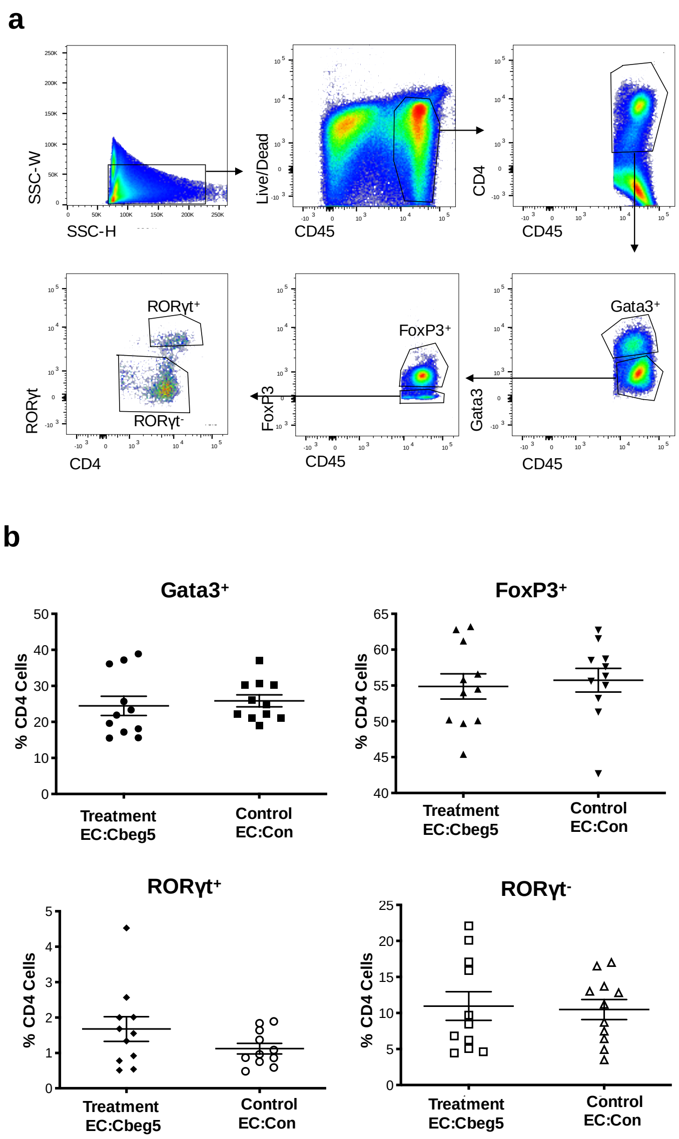

Supplementary Figure 5. Wild-type 8 week C57BL/6 mice were colonized with *E. coli* engineered to express *Cbeg5* (Treatment, EC:Cbeg5) or *E. coli* with an empty vector (Control, EC:Con). After 1 week of colonization the colon LPL were isolated and analyzed by flow cytometry. **a**, After gating for singlets and live cells (Aqua LIVE/DEAD) the gating schema is shown for identification of $CD4^+$ T cell populations based on transcription factors. T cells were gated into 4 populations. **b,** There was no significant difference in T cell populations between the two groups (N = 11 for EC:Cbeg5, N = 11 for EC:Con, error bars are mean +/- s.e.m. Data represents 2 independent experiments, groups compared by unpaired, two sided Mann-Whitney test, $p > 0.05$)

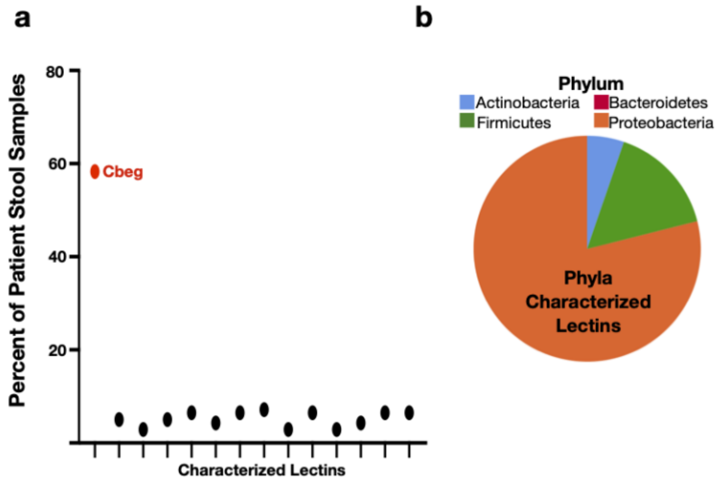

Supplementary Figure 6. A dataset of characterized lectin protein sequences (Unilectin) are searched against human microbiome sequencing datasets to identify highly similar sequences (BLASTP, >90% sequence similarity). **a,** Previously characterized lectins are in a small number of patient stool samples whereas Cbeg4/5 are in a large percentage of patient stool samples. **b,** Characterized lectins are largely from proteobacterial species. No characterized lectins have been identified in Bacteroidetes species from which *Cbeg4* and *Cbeg5* were isolated.

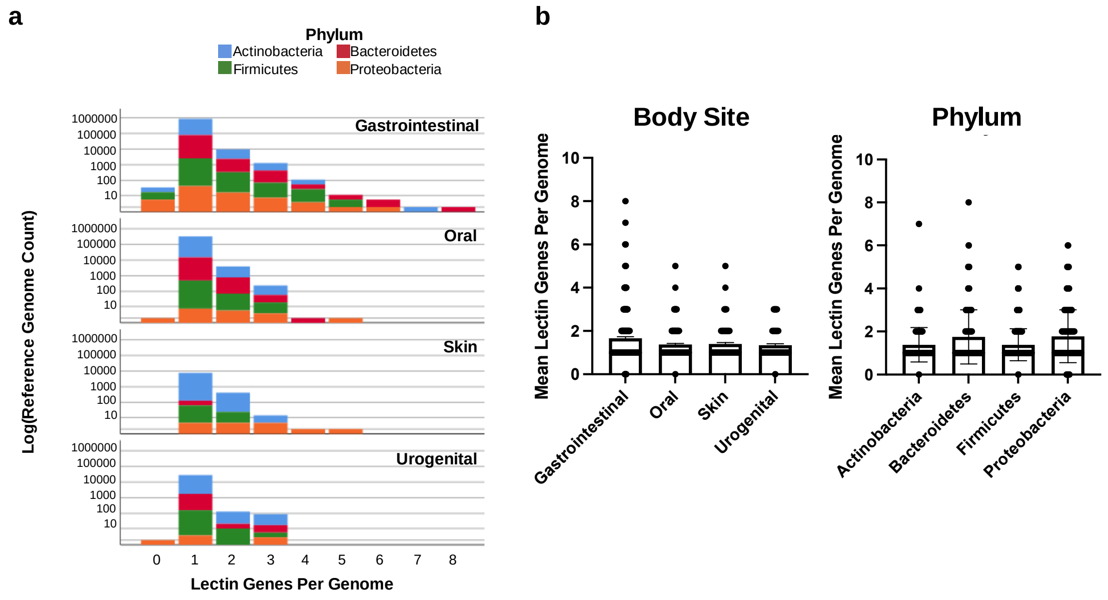

Supplementary Figure 7. a, Human-microbial-lectin dataset was aligned to a database of reference genome sequences generated from bacteria isolated at different sites in the human microbiome (Human Microbiome Project). The number of lectin genes per genome for each bacterial species was determined and plotted as a histogram. Data is presented based on body site from where the bacterium was isolated and is colored by the bacterial phylum. **b,** The mean number of human-microbial-lectin genes per genome is presented per body site and phylum. (Bar graphs are mean +/- s.e.m. , N=240 gastrointestinal, 184 oral, 107 skin, 95 urogential ; N = 403 Firmicutes, 156 Actinobacteria, 152 Proteobacteria, 139 Bacteroidetes)

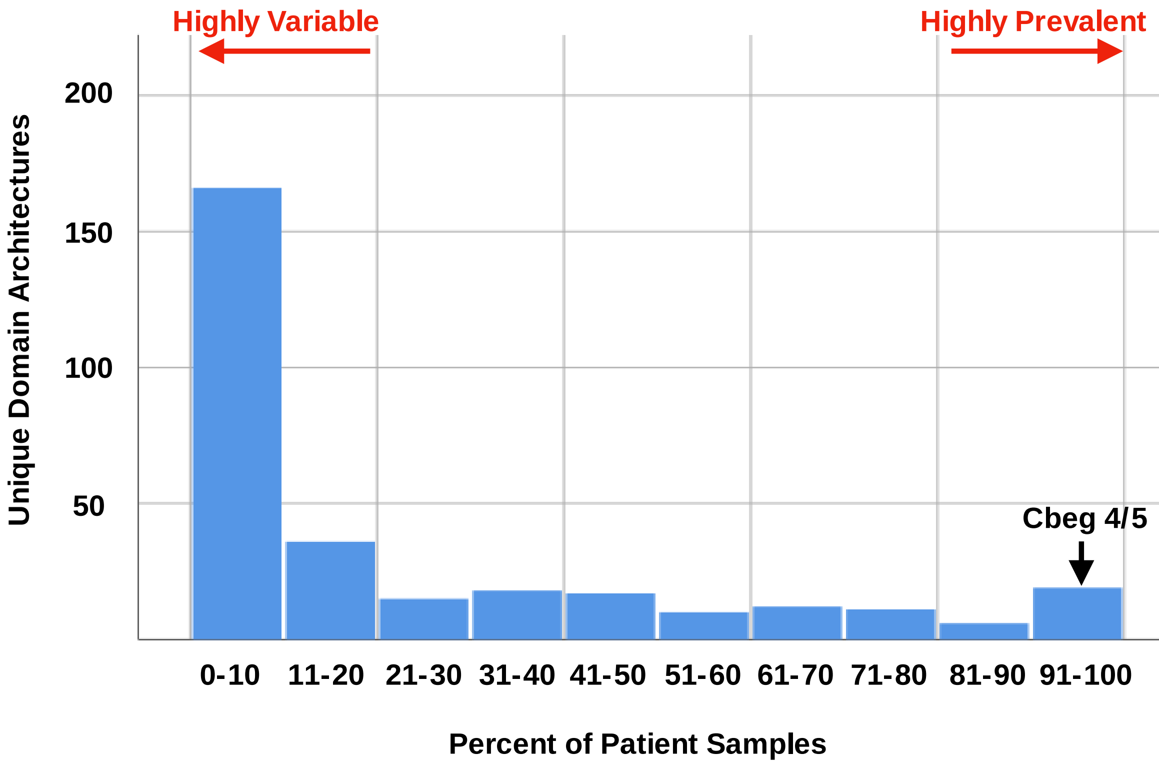

Supplementary Figure 8. In stool samples there are 2278 uncharacterized lectin protein sequences and 314 unique domain architectures. Certain domain architectures are highly prevalent across patient samples being identified in >90% of samples. For most domain architectures there is great variability across patients as they are identified only in <10% of samples. The domain architecture specific to Cbeg4/5 is highly prevalent.

Supplementary Table 1. Antibodies for cytometry (CyTOF, Flow)

Channel	Marker	Clone	Manufacturer	Panel	Dilution
115In	CD11c	Bu15	Biolegend	Cytokine-Cytof	1:100
141Pr	IFNg	B27	Biolegend	Cytokine-Cytof	1:100
142Nd	CD19	HIB19	Biolegend	Cytokine-Cytof	1:100
143Nd	CD45RA	H100	Fluidigm	Cytokine-Cytof	1:100
144Nd	IL-4	MP4-25D2	DVS	Cytokine-Cytof	1:100
145Nd	CD4	RPA-T4	Biolegend	Cytokine-Cytof	1:100
146Nd	CD8	RPA-T8	Biolegend	Cytokine-Cytof	1:100
147Sm	IL-1b	H1b-27	Biolegend	Cytokine-Cytof	1:100
148Nd	CD16	3G8	Biolegend	Cytokine-Cytof	1:100
150Nd	CD1c	L161	Biolegend	Cytokine-Cytof	1:100
151Eu	CD123	6H6	Biolegend	Cytokine-Cytof	1:100
152Sm	CD66b	G10F5	Biolegend	Cytokine-Cytof	1:100
153Eu	TNFa	Mab11	Biolegend	Cytokine-Cytof	1:100
155Gd	CD27	O323	Biolegend	Cytokine-Cytof	1:100
156Gd	IL-6	MQ2-13AS	Fluidigm	Cytokine-Cytof	1:100
158Gd	IL-2	MQ1-17H12	DVS	Cytokine-Cytof	1:100
159Tb	GM-CSF	BVD2-21C11	DVS	Cytokine-Cytof	1:100
160Gd	CD14	M5E2	Biolegend	Cytokine-Cytof	1:100
161Dy	CD56	B159	BD Biosciences	Cytokine-Cytof	1:100
164Dy	IL17A	N49-653	DVS	Cytokine-Cytof	1:100
166Er	IL-10	JES3-9D7	Biolegend	Cytokine-Cytof	1:100
168Er	CD3	UCHT1	Biolegend	Cytokine-Cytof	1:100
170Er	CD38	HB-7	Biolegend	Cytokine-Cytof	1:100
173Yb	IL-8	BH0814	Biolegend	Cytokine-Cytof	1:100
174Yb	HLADR	L243	Biolegend	Cytokine-Cytof	1:100
209Bi	CD11b	ICRF44	Fluidigm	Cytokine-Cytof	1:100
113In	CD57	HCD57	Biolegend	Phospho-Cytof	1:100
115In	CD11c	Bu15	Biolegend	Phospho-Cytof	1:100
142Nd	CD19	HIB19	Biolegend	Phospho-Cytof	1:100
143Nd	CD45RA	HI100	Biolegend	Phospho-Cytof	1:100
145Nd	CD4	RPA-T4	Biolegend	Phospho-Cytof	1:100
146Nd	CD8	RPA-T8	Biolegend	Phospho-Cytof	1:100
148Nd	CD16	3G8	Biolegend	Phospho-Cytof	1:100
149Sm	4ebp1	236B4	Fluidigm	Phospho-Cytof	1:100
150Nd	CD1c	3G8	Biolegend	Phospho-Cytof	1:100
151Eu	CD123	L161	Biolegend	Phospho-Cytof	1:100
152Sm	CD66b	6H6	Biolegend	Phospho-Cytof	1:100
155Gd	CD27	O323	Biolegend	Phospho-Cytof	1:100
156Gd	pp38	D3f9	Fluidigm	Phospho-Cytof	1:100

159Tb	pMAPKAP2	27B7	Fluidigm	Phospho-Cytof	1:100
160Gd	CD14	M5E2	Biolegend	Phospho-Cytof	1:100
161Dy	CD56	B159	BD Biosciences	Phospho-Cytof	1:100
162Dy	p-PLCg2	K86-689.37	Fluidigm	Phospho-Cytof	1:100
164Dy	IkBa	L35A5	Fluidigm	Phospho-Cytof	1:100
165Ho	pCREB	87G3	Fluidigm	Phospho-Cytof	1:100
167Er	pERK	D1314.4E	Fluidigm	Phospho-Cytof	1:100
168Er	CD3	UCHT1	Biolegend	Phospho-Cytof	1:100
170Er	CD38	HB-7	Biolegend	Phospho-Cytof	1:100
174Yb	HLADR	L243	Biolegend	Phospho-Cytof	1:100
175Lu	pS6	N7-548	Fluidigm	Phospho-Cytof	1:100
209Bi	CD11b	ICRF44	Fluidigm	Phospho-Cytof	1:100
Zombie Aqua	Viability	NA	Biolegend	Mouse T Cell + Myeloid	1:1000
APC-Cy7	CD45	30-F11	Biolegend	Mouse T Cell + Myeloid	1:100
APC	CD4	RM4-5	Biolegend	Mouse T Cell	1:100
PacBlue	MHCII	M5/114.15.2	Biolegend	Mouse Myeloid	1:1000
PerCPCCy5.5	CD11b	M1/70	BD Biosciences	Mouse Myeloid	1:1000
PE-Cy7	CD11c	N418	Biolegend	Mouse Myeloid	1:1000
FITC	Ly6C	HK1.4	Biolegend	Mouse Myeloid	1:100
PE	CD64	X54-5/7.1	Biolgened	Mouse Myeloid	1:100
PerCPCCy5.5	RORγt	Q31-378	BD Biosciences	Mouse T Cell	1:100
PE	FoxP3	FJK-16s	eBioScience	Mouse T Cell	1:100
Brilliant Violet 421	Gata3	16E10A23	Biolegend	Mouse T Cell	1:100

Supplementary Table 2. Characterized lectins in the human microbiome

Uniprot ID	Protein names	Organism	Interpro Domain
A0A0V9HGB3	Fimbrial adhesin (Fimbrial protein) (Putative fimbrial-like exported adhesin protein)	Escherichia coli	IPR036937;IPR008966;IPR015243;
A0A140UH97	FimG protein	Escherichia coli O6:K15:H31 (strain 536 / UPEC)	
A0A182DW20	PapG, lectin domain	Escherichia coli	IPR008966;IPR005310;IPR038420;
A0A2J7MV87	Fimbrial adhesin (Fimbrial protein) (Fimbrial-like adhesin protein) (Putative fimbrial-like adhesin protein)	Escherichia coli	IPR036937;IPR008966;IPR015243;
A3CM52	Platelet-binding glycoprotein	Streptococcus sanguinis (strain SK36)	IPR019948;IPR022263;IPR019931;IPR026465;
O52269	Adhesin binding fucosylated histo-blood group antigen	Helicobacter pylori (Campylobacter pylori)	IPR002718;IPR040838;
P08189	Protein FimF	Escherichia coli (strain K12)	IPR000259;IPR036937;IPR008966;
P08190	Protein FimG	Escherichia coli (strain K12)	IPR036937;IPR008966;
P08191	Type 1 fimbrin D-mannose specific adhesin (Protein FimH)	Escherichia coli (strain K12)	IPR000259;IPR036937;IPR008966;IPR015243;
P31697	Chaperone protein FimC	Escherichia coli (strain K12)	IPR013783;IPR008962;IPR036316;IPR001829;IPR016148;IPR018046;IPR016147;
Q2PHL4	Thiol-activated cytolysin	Streptococcus mitis	IPR000421;IPR006585;IPR008979;IPR035390;IPR038700;IPR001869;IPR036363;IPR036359;
Q47450	Uncharacterized protein	Escherichia coli	IPR036937;IPR008966;IPR005310;IPR038420;IPR005309;
Q7U0N6	Fumarate hydratase class II (Fumarase C) (EC 4.2.1.2) (Aerobic fumarase) (Iron-independent fumarase)	Mycobacterium bovis (strain ATCC BAA-935 / AF2122/97)	IPR005677;IPR024083;IPR018951;IPR020557;IPR000362;IPR022761;IPR008948;
Q939N5	Platelet binding protein GspB (Adhesin GspB) (Serine-rich adhesin for platelets) (Serine-rich repeat protein GspB)	Streptococcus gordonii	IPR019948;IPR022263;IPR019931;IPR026465;
Q9AP05	FimH	Escherichia coli	IPR000259;IPR036937;IPR008966;IPR015243;
Q9AYY6	Tail spike protein	Enterobacteria phage HK620 (Bacteriophage HK620)	IPR009093;IPR036730;IPR012334;IPR011050;IPR041303;
Q9F5R9	FimH	Escherichia coli	IPR036937;IPR008966;IPR015243;
Q9F6Z7	FimH (FimH protein) (Fimbrial protein) (Minor component of type 1 fimbriae) (Protein FimH) (Type 1 fimbria D-mannose specific adhesin FimH) (Type 1 fimbriae D-mannose specific adhesin FimH) (Type 1 fimbrial adhesin) (Type 1 fimbrial adhesin subunit FimH)	Escherichia coli	IPR000259;IPR036937;IPR008966;IPR015243;
Q9S497	FimH	Escherichia coli	IPR000259;IPR036937;IPR008966;IPR015243;
Q9ZKV2	Outer membrane protein-adhesin	Helicobacter pylori (strain J99 / ATCC 700824) (Campylobacter pylori J99)	IPR002718;IPR040838;

Supplementary Table 3. Carbohydrate binding domains from Interpro

Interpro ID	Description	Interpro ID	Description
IPR000254	Cellulose-binding domain, fungal	IPR009011	Mannose-6-phosphate receptor binding domain superfamily
IPR000296	Cation-dependent mannose-6-phosphate receptor	IPR009031	Carbohydrate binding module family 10
IPR000479	Cation-independent mannose-6-phosphate receptor	IPR009342	Putative carbohydrate binding domain
IPR000772	Ricin B, lectin domain	IPR009960	Fungal fruit body lectin
IPR000922	D-galactoside/L-rhamnose binding SUEL lectin domain	IPR010502	Carbohydrate-binding domain, family 9
IPR000985	Legume lectin, alpha chain, conserved site	IPR010907	Calcium-mediated lectin
IPR001079	Galectin, carbohydrate recognition domain	IPR011611	Carbohydrate kinase PfkB
IPR001101	Plectin repeat	IPR012111	Hemolectin/hemocytin
IPR001220	Legume lectin domain	IPR012291	CBM2, carbohydrate-binding domain superfamily
IPR001229	Jacalin-like lectin domain	IPR012475	Fucose-specific lectin
IPR001304	C-type lectin-like	IPR013320	Concanavalin A-like lectin/glucanase domain superfamily
IPR001480	Bulb-type lectin domain	IPR013784	Carbohydrate-binding-like fold
IPR001538	Mannose-6-phosphate isomerase, type II, C-terminal	IPR014718	Glycoside hydrolase-type carbohydrate-binding
IPR001732	UDP-glucose/GDP-mannose dehydrogenase, N-terminal	IPR015243	FimH, mannose-binding domain
IPR001919	Carbohydrate-binding type-2 domain	IPR015295	Carbohydrate binding module 27
IPR001956	Carbohydrate-binding module 3	IPR015303	Fimbrial adhesin F17-AG, lectin domain
IPR002044	Carbohydrate binding module family 20	IPR015344	Vibrio cholerae neuraminidase, lectin-like domain
IPR002396	Selectin superfamily	IPR015533	Galectin-4/6
IPR002883	CBM10/dockerin domain	IPR015534	Galectin-3
IPR002889	Carbohydrate-binding WSC	IPR015926	Cytolysin/lectin
IPR003305	Carbohydrate-binding, CenC-like	IPR016186	C-type lectin-like/link domain superfamily
IPR003610	Carbohydrate-binding module family 5/12	IPR016187	C-type lectin fold
IPR004302	Cellulose/chitin-binding protein, N-terminal	IPR016305	Mannose-6-phosphate isomerase
IPR004954	Putative mucin/carbohydrate-binding domain	IPR016348	L-selectin
IPR005036	CBM21 (carbohydrate binding type-21) domain	IPR016363	Legume lectin
IPR005052	Legume-like lectin	IPR016817	Mannose-P-dolichol utilization defect 1 protein
IPR005084	Carbohydrate binding module family 6	IPR016927	Lectin, sugar-binding
IPR005085	Carbohydrate binding module family 25	IPR018366	Carbohydrate-binding type-2, conserved site
IPR005086	Carbohydrate binding module family 17/28	IPR018378	C-type lectin, conserved site
IPR005087	Carbohydrate binding module family 11	IPR018909	Endo-1,3(4)-beta-glucanase 1, carbohydrate binding
IPR005088	Carbohydrate binding module family 15	IPR031798	Alginate biosynthesis protein AlgX, C-terminal carbohydrate-binding module
IPR005089	Carbohydrate-binding module family 19	IPR032496	Hemolysin, beta-prism lectin
IPR005102	Carbohydrate binding X2 domain	IPR032798	Endoglucanase Z, cellulose-binding domain
IPR005310	PapG, carbohydrate-binding domain	IPR033734	Jacalin-like lectin domain, plant
IPR005323	Pullulanase, carbohydrate-binding module 41	IPR033790	Lectin Bc2l-C, N-terminal
IPR006584	Cellulose binding, type IV	IPR033801	CBM6/CBM35/CBM36-like 1
IPR006585	Fucolectin tachylectin-4 pentraxin-1	IPR033802	Carbohydrate-binding protein CttA, X module
IPR006633	Carbohydrate-binding/sugar hydrolysis domain	IPR033803	CBM6/CBM35/CBM36-like 2
IPR007117	Expansin, cellulose-binding-like domain	IPR033816	Eosinophil major basic protein, C-type lectin-like domain
IPR008965	CBM2/CBM3, carbohydrate-binding domain superfamily	IPR033862	Putative metalloprotease, Jacalin-like lectin domain
IPR019019	H-type lectin domain	IPR033987	Aggrecan/versican, C-type lectin-like domain
IPR019028	Carbohydrate binding domain CBM49	IPR033988	CEL-1-like C-type lectin-like domain
IPR019825	Legume lectin, beta chain, Mn/Ca-binding site	IPR033989	CD209-like, C-type lectin-like domain
IPR021720	Malectin domain	IPR033990	Collectin, C-type lectin-like domain
IPR022386	Carbohydrate ABC transporter, N-acetylglucosamine/diacetylchitobiose-binding protein	IPR033991	Selectin, C-type lectin-like domain
IPR022387	Carbohydrate ABC transporter substrate-binding, CPR0540	IPR033992	Natural killer cell receptor-like, C-type lectin-like domain
IPR023294	Tachylectin 2	IPR034007	C-type lectin-like domain, bacterial
IPR024361	Bacteroidetes-Associated Carbohydrate-binding Often N-terminal	IPR034008	TC14-like, C-type lectin-like domain
IPR024691	Micronemal protein 1, galectin-like domain	IPR034010	DGCR2-like, C-type lectin-like domain
IPR024788	Malectin-like domain	IPR034011	Attractin-like, C-type lectin-like domain
IPR025112	Putative carbohydrate metabolism domain	IPR034831	Laforin, CBM20 domain
IPR025584	Carbohydrate-binding domain-containing protein Cthe_2159	IPR034835	Beta-amylase, CBM20 domain
IPR026195	P-selectin glycoprotein ligand 1	IPR034836	Glucoamylase, CBM20 domain
IPR027613	Surface carbohydrate biosynthesis protein, LIC13510 family	IPR034838	Starch-binding domain-containing protein 1, CBM20 domain
IPR028927	Mannose-6-phosphate receptor	IPR034839	Glycerophosphocholine phosphodiesterase GPCPD1, CBM20 domain
IPR028988	Hemolytic lectin CEL-III, C-terminal domain superfamily	IPR034840	DPE2, CBM20 domain 1

IPR029665	GDP-mannose transporter GONST3, plant
IPR030269	Plectin
IPR030638	Galectin-8
IPR030640	Galectin-7
IPR030649	Galectin-12
IPR030650	Galectin-16
IPR030651	Galectin-related protein
IPR030652	Galectin-14
IPR030653	Galectin-13
IPR035992	Ricin B-like lectins
IPR036404	Jacalin-like lectin domain superfamily
IPR036426	Bulb-type lectin domain superfamily
IPR036573	Carbohydrate-binding module superfamily 5/12
IPR036601	CBM10 superfamily
IPR036684	Calcium-mediated lectin superfamily
IPR036749	Expansin, cellulose-binding-like domain superfamily
IPR036813	Tachylectin 2 superfamily
IPR036966	Carbohydrate-binding module 3 superfamily
IPR037221	H-type lectin domain superfamily
IPR037570	Mannose-binding protein C
IPR037571	Mannan-binding lectin-associated serine peptidase 2
IPR038175	CBM21 domain superfamily
IPR038420	PapG, carbohydrate-binding domain superfamily
IPR038560	Beta-1,3-xylanase, CBM31 domain superfamily
IPR038637	NPCBM domain superfamily
IPR038653	Putative carbohydrate metabolism domain superfamily
IPR038736	GDP-mannose transporter Vrg4-like
IPR039155	Malectin
IPR040249	Ricin B-like lectin EULS3-like
IPR040528	Lectin-like domain
IPR040669	Agarase, CBM-like domain
IPR040877	Carbohydrate binding module 65 domain 1
IPR040946	Endoglucanase B, carbohydrate binding domain
IPR040959	Cellulose-binding protein CttA, N-terminal
IPR040964	OAA-family lectin sugar binding domain
IPR041253	Carbohydrate binding module 77
IPR041438	Carbohydrate-binding module 64
IPR041447	Mannosidase Ig/CBM-like domain
IPR042129	Mannose-binding protein A
IPR042190	Killer cell lectin-like receptor subfamily G member 1

IPR034841	DPE2, CBM20 domain 2
IPR034849	Maltogenic alpha-amylase, CBM20 domain
IPR035310	Galactose-inhibitable lectin 35kDa subunit
IPR035661	EMP46/EMP47, N-terminal lectin domain
IPR035664	VIP36, lectin domain
IPR035665	VcfQ, lectin domain
IPR035971	Cellulose-binding domain superfamily
IPR035992	Ricin B-like lectins

Supplementary Table 4. Human-microbial-lectin distribution in body sites

Body Site(s)	total		Body Site(s)	total
Stool	2158		Buccal Gingiva Palatine Tonsils R Retroauricular Saliva Subgingival Plaque Supragingival Plaque Throat Tongue	2
Supragingival Plaque	64		Buccal Gingiva Palatine Tonsils Subgingival Plaque Supragingival Plaque Throat Tongue	2
Buccal Stool	57		Buccal Gingiva Saliva Subgingival Plaque Supragingival Plaque Tongue	2
Buccal Subgingival Plaque Supragingival Plaque Tongue	26		Buccal Gingiva Subgingival Plaque Supragingival Plaque Throat Tongue	2
Subgingival Plaque Supragingival Plaque	24		Buccal Palatine Tonsils Saliva Subgingival Plaque Supragingival Plaque Tongue	2
Supragingival Plaque Tongue	23		Buccal Saliva Subgingival Plaque Supragingival Plaque Tongue	2
Posterior Fornix	22		Palatine Tonsils Saliva Throat Tongue	2
Tongue	21		Palatine Tonsils Subgingival Plaque Supragingival Plaque Throat	2
Buccal Palatine Tonsils Supragingival Plaque Throat Tongue	20		Subgingival Plaque Supragingival Plaque Throat Tongue	2
Stool Subgingival Plaque	20		Gingiva Palatine Tonsils Saliva	2
Buccal Palatine Tonsils Saliva Subgingival Plaque Supragingival Plaque Throat Tongue	18		Buccal Saliva Throat	2
Buccal Gingiva Subgingival Plaque Supragingival Plaque Tongue	14		Buccal Throat Tongue	2
Palatine Tonsils Supragingival Plaque Throat Tongue	14		Subgingival Plaque Throat Tongue	2
Subgingival Plaque Supragingival Plaque Tongue	14		Buccal Subgingival Plaque	2
L Retroauricular Stool	14		Saliva Stool	2
Buccal Palatine Tonsils Subgingival Plaque Supragingival Plaque Throat Tongue	13		L Retroauricular	2
Buccal Gingiva Palatine Tonsils Saliva Subgingival Plaque Supragingival Plaque Throat Tongue	12		R Retroauricular	2
Buccal Supragingival Plaque Tongue	12		Throat	2
Buccal	11		Anterior Nares Buccal Gingiva Palatine Tonsils R Retroauricular Saliva Subgingival Plaque Supragingival Plaque Throat Tongue	1
Saliva	11		Buccal Gingiva L Retroauricular Palatine Tonsils R Retroauricular Saliva Subgingival Plaque Supragingival Plaque Throat Tongue	1
Buccal Palatine Tonsils Saliva Supragingival Plaque Throat Tongue	10		Anterior Nares Buccal Gingiva Palatine Tonsils R Retroauricular Supragingival Plaque Throat Tongue	1
Vaginal Introitus	9		Buccal Palatine Tonsils Saliva Stool Subgingival Plaque Supragingival Plaque Throat Tongue	1
Palatine Tonsils Subgingival Plaque Supragingival Plaque	8		Buccal Gingiva Palatine Tonsils Saliva Subgingival Plaque Supragingival Plaque Tongue	1
Subgingival Plaque	8		Buccal Palatine Tonsils Saliva Stool Subgingival Plaque Supragingival Plaque Throat	1
Buccal Palatine Tonsils Saliva Throat Tongue	7		Buccal Gingiva Palatine Tonsils Saliva Supragingival Plaque Tongue	1
Palatine Tonsils Subgingival Plaque Supragingival Plaque Throat Tongue	7		Buccal Gingiva Palatine Tonsils Supragingival Plaque Throat Tongue	1
Buccal Supragingival Plaque Throat Tongue	7		Buccal Gingiva Saliva Supragingival Plaque Throat Tongue	1
Mid Vagina Vaginal Introitus	7		Buccal Gingiva Palatine Tonsils Subgingival Plaque Supragingival Plaque	1
Stool Supragingival Plaque	7		Buccal Gingiva Saliva Supragingival Plaque Tongue	1
Supragingival Plaque Throat Tongue	6		Buccal Palatine Tonsils Saliva Stool Throat	1
Anterior Nares Buccal Gingiva Palatine Tonsils Saliva Supragingival Plaque Throat Tongue	5		Buccal Palatine Tonsils Stool Throat Tongue	1
Buccal Subgingival Plaque Supragingival Plaque	5		Buccal Palatine Tonsils Subgingival Plaque Supragingival Plaque Throat	1
Buccal Gingiva Palatine Tonsils Saliva Supragingival Plaque Throat Tongue	4		Buccal Palatine Tonsils Subgingival Plaque Throat Tongue	1
Buccal Gingiva Supragingival Plaque Tongue	4		Palatine Tonsils Stool Supragingival Plaque Throat Tongue	1
Palatine Tonsils Supragingival Plaque Tongue	4		Anterior Nares Supragingival Plaque Throat Tongue	1
Palatine Tonsils Throat Tongue	4		Buccal Palatine Tonsils Supragingival Plaque Tongue	1
Saliva Subgingival Plaque Supragingival Plaque	4		Buccal Saliva Stool Throat	1

Throat Tongue	4		Palatine Tonsils Saliva Supragingival Plaque Tongue	1
Mid Vagina	4		Buccal Gingiva Palatine Tonsils	1
Buccal Palatine Tonsils Subgingival Plaque Supragingival Plaque Tongue	3		Buccal Gingiva Supragingival Plaque	1
Buccal Subgingival Plaque Supragingival Plaque Throat Tongue	3		Gingiva Palatine Tonsils Subgingival Plaque	1
Palatine Tonsils Saliva Subgingival Plaque Supragingival Plaque Tongue	3		Buccal Stool Subgingival Plaque	1
Buccal Palatine Tonsils Throat Tongue	3		Buccal Stool Tongue	1
Palatine Tonsils Subgingival Plaque Supragingival Plaque Tongue	3		Buccal Subgingival Plaque Tongue	1
Anterior Nares L Retroauricular R Retroauricular	3		L Retroauricular Stool Tongue	1
Mid Vagina Posterior Fornix Vaginal Introitus	3		Saliva Subgingival Plaque Throat	1
Stool Supragingival Plaque Tongue	3		Saliva Supragingival Plaque Tongue	1
Subgingival Plaque Supragingival Plaque Throat	3		Stool Throat Tongue	1
Buccal Supragingival Plaque	3		Buccal Gingiva	1
Buccal Tongue	3		Gingiva Palatine Tonsils	1
L Retroauricular R Retroauricular	3		Palatine Tonsils Supragingival Plaque	1
Palatine Tonsils Tongue	3		Palatine Tonsils Throat	1
Stool Throat	3		R Retroauricular Stool	1
Gingiva	3		Stool Tongue	1
Palatine Tonsils	3		Stool Vaginal Introitus	1
			Subgingival Plaque Throat	1
			Anterior Nares	1

Supplementary Table 5. Unique human-microbial-lectins per body site

Body Site	Total Lectin Genes Per Body Site	Lectin Genes Specific to Body Site	Percent Unique Lectin Genes in Each Body Site
Anterior Nares	12	1	8
Gingiva	69	3	4
Buccal	282	11	4
L Retroauricular	26	2	8
Mid Vagina	14	4	28
Palatine Tonsil	179	3	2
Posterior Fornix	25	22	88
Saliva	105	11	10
Stool	2278	2158	95
R Retroauricular	14	2	14
Subgingival plaque	224	8	4
Supragingival plaque	384	64	17
Throat	183	2	1
Tongue	316	21	7
Vaginal introitus	20	9	45
Total	2807*	2321	

***** It is not the sum of the column which would include protein sequences found at multiple body sites but the total unique protein sequences across all body sites.

Supplementary Table 6. Cloning primers

Protein Variant	Subcloning Primers [a]	Restriction Sites
His$_6$-Cbeg5$^{21–957}$	F: GAGACATATGCAAAGTCTGGAACAGACCACAG	NdeI
	R: GAGACTCGAG**CTA**CCGCCGTTTTTTGGACAAC	XhoI
His$_6$-Cbeg5-FN	F: GAGACATATGCCGCTTCCGGTGAATCACT	NdeI
	R: GAGACTCGAG**CTA**ATAGGCAGACAGAATTTCGGAAGG	XhoI
GFP-Cbeg5-CBM	F: CTGAGCGGCCGCGTGTTTACGCCAC	NotI
	R: CTGACTCGAGGCCCCGCACAATGTT	XhoI
GFP-Cbeg4-CBM	F: CTGAGCGGCCGCGTTTATACCCCTCG	NotI
	R: CTGACTCGAGACCGCGCGATAT	XhoI

[a] Restriction site is underlined. Stop codon is bolded.
