# Supplementary material for: Unraveling function and diversity of bacterial lectins in the human microbiome
Source: Nat Commun. 2022 Jun 3;13:3101. doi: 10.1038/s41467-022-29949-3 (PMC9166713; doi:10.1038/s41467-022-29949-3)
Supplement: Supplementary file 6 — Reporting Summary [file 41467_2022_29949_MOESM6_ESM.pdf]

nature research

Corresponding author(s): Louis Cohen, Sean Brady

Last updated by author(s): Nov 15, 2021

Reporting Summary

Nature Research wishes to improve the reproducibility of the work that we publish. This form provides structure for consistency and transparency in reporting. For further information on Nature Research policies, see our Editorial Policies and the Editorial Policy Checklist.

Statistics

For all statistical analyses, confirm that the following items are present in the figure legend, table legend, main text, or Methods section.

n/a	Confirmed	
☐	☒	The exact sample size (*n*) for each experimental group/condition, given as a discrete number and unit of measurement
☐	☒	A statement on whether measurements were taken from distinct samples or whether the same sample was measured repeatedly
☐	☒	The statistical test(s) used AND whether they are one- or two-sided<br>*Only common tests should be described solely by name; describe more complex techniques in the Methods section.*
☒	☐	A description of all covariates tested
☐	☒	A description of any assumptions or corrections, such as tests of normality and adjustment for multiple comparisons
☐	☒	A full description of the statistical parameters including central tendency (e.g. means) or other basic estimates (e.g. regression coefficient) AND variation (e.g. standard deviation) or associated estimates of uncertainty (e.g. confidence intervals)
☐	☒	For null hypothesis testing, the test statistic (e.g. *F*, *t*, *r*) with confidence intervals, effect sizes, degrees of freedom and *P* value noted<br>*Give P values as exact values whenever suitable.*
☒	☐	For Bayesian analysis, information on the choice of priors and Markov chain Monte Carlo settings
☒	☐	For hierarchical and complex designs, identification of the appropriate level for tests and full reporting of outcomes
☒	☐	Estimates of effect sizes (e.g. Cohen's *d*, Pearson's *r*), indicating how they were calculated

Our web collection on statistics for biologists contains articles on many of the points above.

Software and code

Policy information about availability of computer code

Data collection	NA
Data analysis	There is no unique code used in this paper. Existing programs are cited in methods including websites for download when available. Programs used include Cytobank, ProcartaPlex Analyst 1.0, Flow v 10.8.1, Prism V9

For manuscripts utilizing custom algorithms or software that are central to the research but not yet described in published literature, software must be made available to editors and reviewers. We strongly encourage code deposition in a community repository (e.g. GitHub). See the Nature Research guidelines for submitting code & software for further information.

Data

Policy information about availability of data

All manuscripts must include a data availability statement. This statement should provide the following information, where applicable:
- Accession codes, unique identifiers, or web links for publicly available datasets
- A list of figures that have associated raw data
- A description of any restrictions on data availability

All figure data is available in the source data sheet. Gene accession numbers for all cloned genes are provided in methods. Publicly available DNA and RNA datasets analyzed in this study are referenced accordingly and references contain links to datasets available for download. Datasets used include Uniprot, Glyconnect, Interpro.

Field-specific reporting

Please select the one below that is the best fit for your research. If you are not sure, read the appropriate sections before making your selection.

☒ Life sciences ☐ Behavioural & social sciences ☐ Ecological, evolutionary & environmental sciences

For a reference copy of the document with all sections, see nature.com/documents/nr-reporting-summary-flat.pdf

Life sciences study design

All studies must disclose on these points even when the disclosure is negative.

Sample size	Sample sizes were not predetermined as this was an exploratory analysis. It was aimed to have at least 12 mice per treatment group for the analysis which is sufficiently powered to detect a 10% difference between groups in endpoints with a 5% standard deviation. At 15 mice per group we had more then sufficient power to detect the observed differences in our endpoints.
Data exclusions	Data was not excluded from the manuscript
Replication	All animal experiments were performed with at least three replicates (individual mice) per treatment group and with at least 2 independent experiments. Analysis of intestinal leukocyte populations (Fig 3) was performed with 4 independent experiments. Experiments involving human cells (PBMC and intestinal leukocytes) were performed with at least 2 independent experiments from separate individuals. All attempts at replication were successful.
Randomization	Cages were randomly allocated to treatment groups . There was no predetermined randomization strategy. As the only variable was treatment group covariates did not need to be adjusted. Sex, age and weight were equal between groups as all mice were used shortly after receipt from Jackson Labs.
Blinding	Pathologists were blinded during the collection and analysis of samples. The researchers were not blinded to the mouse treatment groups to ensure tracking of the correct microbes being administered to the animals and to ensure downstream applications could be applied correctly.

Reporting for specific materials, systems and methods

We require information from authors about some types of materials, experimental systems and methods used in many studies. Here, indicate whether each material, system or method listed is relevant to your study. If you are not sure if a list item applies to your research, read the appropriate section before selecting a response.

Materials & experimental systems

n/a	Involved in the study	
☐	☒	Antibodies
☒	☐	Eukaryotic cell lines
☒	☐	Palaeontology and archaeology
☐	☒	Animals and other organisms
☐	☒	Human research participants
☒	☐	Clinical data
☒	☐	Dual use research of concern

Methods

n/a	Involved in the study	
☒	☐	ChIP-seq
☐	☒	Flow cytometry
☒	☐	MRI-based neuroimaging

Antibodies

Antibodies used	All antibodies are cited in Supplementary Table 1
Validation	All antibodies were purchased from established manufacturers and validated by the manufacturer. The manufacturers and clones are provided in Supplementary Table 1. Antibody validation by the manufacturer includes structural and functional validation as reviewed for each antibody on the technical data sheet. No antibodies used in this project have not been validated or previously published on. Each antibody validaiton is available on the manufacturers website.

Animals and other organisms

Policy information about studies involving animals; ARRIVE guidelines recommended for reporting animal research

Laboratory animals	8 week, male and female C57BL/6 mice. Mice were housed at specified pathogen free (SPF) health status in individually ventilated cages at 21-22°C and 39%–50% humidity. Mice light/dark cycle is 12 hours light and 12 hours dark 7 days a week.
Wild animals	None

Field-collected samples	None
Ethics oversight	Mount Sinai IACUC. Protocol referenced in manuscript.

Note that full information on the approval of the study protocol must also be provided in the manuscript.

Human research participants

Policy information about studies involving human research participants

Population characteristics	Healthy patients are recruited at Mount Sinai to provide blood for PBMC isolation (IRB protocol number provided in methods). Patients undergoing colon resection are recruited at Mount Sinai to provide tissue (IRB protocol number provided in methods). All patients are consented. No specific age, gender or clinical characteristics were required by the investigators for PBMC use. For colon resection samples patients were required to not have immune diseases of the intestine or be taking immune suppressing medications.
Recruitment	Healthy patients have previously agreed to provide PBMC at Mount Sinai and can be contacted for future donations. Patients undergoing a colon resection are recruited by their surgeon prior to surgery. Discarded tissue specimens are used for cell isolation.
Ethics oversight	Mount Sinai Institutional Review Board.

Note that full information on the approval of the study protocol must also be provided in the manuscript.

Flow Cytometry

Plots

Confirm that:

- [x] The axis labels state the marker and fluorochrome used (e.g. CD4-FITC).
- [x] The axis scales are clearly visible. Include numbers along axes only for bottom left plot of group (a 'group' is an analysis of identical markers).
- [x] All plots are contour plots with outliers or pseudocolor plots.
- [x] A numerical value for number of cells or percentage (with statistics) is provided.

Methodology

Sample preparation	For flow cytometry: For analysis by flow cytometry 100 µL of 2.4G2 Fc blocker was added to cells and incubated at 4°c for 10 min. Cells were then centrifuged at 2000rpm for 2min and resuspended in FACS buffer with antibodies (Supplementary Table 1). Antibodies stained for 30 min protected from light at 4 °C. After staining cells were fixed with 2% PFA for 15 min at room temperature. Cells were then washed with FACS buffer three times and analyzed on the LSRII.<br><br>For CyTOF: Cells were washed twice with FACS buffer (DPBS w 3% FBS and 1 mM EDTA) and fixed in 1.6% paraformaldehyde for 20 minutes at room temperature. Cells were then washed twice and diluted in FACS buffer and placed at 4 °C until staining. Prior to CyTOF assays previously optimized antibody mixtures were prepared in cell staining media (CSM, Fluidigm). Antibody lists are outlined in Supplementary Table 1. Each sample was washed and resuspended in 800 µl of 1X Barcode Perm Buffer (Fluidigm Inc.). Compatible Pd-barcodes were thawed, resuspended in 100 µL of 1X Barcode Perm Buffer and added to the samples. Samples were incubated on ice for 30 minutes then washed in CSM and pooled together. Each barcoded set of samples (corresponding to a single treatment condition) was first resuspended in 100 µL of CSM containing 100 U ml-1 heparin (Sigma) to block non-specific MaxPar Antibody binding. A titrated surface antibody panel designed to allow identification of all major immune subsets (Supplementary Table 1) was prepared in an additional 100 µl of CSM, filtered through a 0.1 µm spin filter (Amicon) and added directly to the sample. Samples were stained for 30 minutes on ice, then washed with CSM and fixed with freshly diluted 2% formaldehyde (Electron Microscopy Sciences) in PBS to cross-link and preserve all surface antibodies. The samples were then washed and permeabilized by the addition of 1 ml of ice-cold 100% methanol, added dropwise while vortexing. Samples were incubated on ice for 30 minutes (or transferred to −80 °C for long term storage), after which they were washed twice with CSM and again resuspended in 100 ul of CSM containing 100 U ml-1 heparin.
Instrument	LSRII, CyTOF
Software	Flowjo and Cytobank using standard methods.
Cell population abundance	No sorting
Gating strategy	Gating strategy provided in the manuscript and in the extended data.<br><br>For PBMC CyTOF assays<br>All cells are gated for singlets based on DNA staining.<br>B cells are CD19+CD3-.<br>T Cells are CD3+ CD19- then CD8+CD4- (CD8 T Cells), CD4+CD8- (CD4 T Cells) or CD4-CD8- (CD4-CD8- T Cells).<br>NK Cells are CD3-CD19-HLADR-CD56+.

Neutrophils are CD66b+, HLADR-, CD56-, CD3-, CD19-.
CD14 monocytes are HLADR+CD56-CD66b-CD14+CD16-CD11c+CD1c-CD123-CD3-CD19-.
CD16 monocytes are HLADR+CD56-CD66b-CD14-CD16+CD11c+CD1c-CD123-CD3-CD19-
mCD11c Myeliod Cells are HLADR+CD56-CD66b-CD14-CD16-CD11c+CD1c-CD123-CD3-CD19-
cDC2 Dendritic Cells are HLADR+CD56-CD66b-CD14-CD16-CD11c+CD1c+CD123-CD3-CD19-
plasmaDC are HLADR+CD56-CD66b-CD14-CD16-CD11c-CD11b-CD1c-CD123+CD3-CD19-
Macrophages are MHCII+CD11c+CD64+CD11b+
Monocytes are MHCII-Ly6c+CD11b+
For Colon Leukocytes
All cells are gated Live Dead
Cd1c+ cells are Cd3-CD19-, HLADR+, CD1c+
CD14+ cells are CD3-CD19-HLADR+CD14+CD16-

For Mouse Experiments
All cells are gated Live Dead and CD45+
Gata3+ cells are CD4+Gata3+
FoxP3 cells are CD4+Gata3-FoxP3+
RORyt cells are CD4+Gata3-FoxP3-RORyt+

☒ Tick this box to confirm that a figure exemplifying the gating strategy is provided in the Supplementary Information.
